# A review of national public health strategies in selected countries

**DOI:** 10.3389/fpubh.2025.1391795

**Published:** 2025-03-18

**Authors:** Michelle Norris, Eimear Burke, Fearghal Comaskey, Máire A. Connolly, Yvonne Doyle, Carol McLoughlin, Leah McManus, Valerie Power, Susan Spillane, Máirín Ryan, Michelle O’Neill

**Affiliations:** ^1^Health Information and Quality Authority, Smithfield, Dublin, Ireland; ^2^Public Health Area E, Health Service Executive (HSE), Limerick, Ireland; ^3^School of Health Sciences, College of Medicine, Nursing and Health Sciences, University of Galway, Galway, Ireland; ^4^Research Department of Epidemiology and Public Health, University College London, London, United Kingdom; ^5^Department of Pharmacology & Therapeutics, Trinity College Dublin, Trinity Health Sciences, Dublin, Ireland

**Keywords:** health promotion, health improvement, health intelligence, health protection, frameworks, plans, public health, strategies

## Abstract

National public health strategies play an essential role in defining a country’s vision, policy direction and approaches for ensuring the health of its population. Gaining an understanding of the public health issues which countries prioritize, and how these health issues are improved upon, may provide insights into effective methods of national public health strategy development and delivery. National public health strategies were identified for ten countries and a descriptive analysis of strategy contents and development was undertaken. All of the identified national public health strategies included an overall aim of improving health and wellbeing, and all strategy scopes extended beyond the health domain. Themes or priorities frequently identified included promoting healthy living or behaviors, the strengthening of public health, and equity or equality. Approaches to strategy implementation were considered to be prescriptive or interpretive, with prescriptive approaches including detailed implementation plans with specific actions and key performance indicators, and interpretive approaches including high-level strategic actions along with principles to support their implementation. National public health strategy development was informed by both evidence and engagement with stakeholders, including the public, experts, and representatives from governmental and non-governmental organizations at local, regional and national levels. Overall, while the information gained in this analysis assists in the development of a national public health strategy, variation observed across the included strategies indicates that consideration should be given to factors which vary from country to country. These factors include the national health profile, the political context, underpinning and associated policies, and the availability of resources.

## 1 Introduction

A national public health strategy plays an essential role in defining a country’s vision and framework for improving national population health outcomes (1). It serves as a road map for a national government to align efforts, resources and policies to improve the health and well-being of the population, prevent diseases, reduce health inequalities, and respond effectively to emerging health threats and public health emergencies (1). All encompassing, the broad goals of national public health strategies may be matched in breadth by strategy scope, with strategies often presented as multisectoral, and addressing not only healthcare, but also the wider determinants of health and health inequalities (2).

Given this broad scope, and the multiple factors which may influence population health, such as the national health situation, underpinning national policies, the health system structure and the availability of resources (3), each country must undergo priority setting to identify the key health issues to focus on within a certain time period (3). These priorities form the basis of any given national public health strategy, and can vary from country to country, and over time. For example, previously, issues related to the environment and climate may have been siloed within environmental strategies. However, more recently the significant impact of climate change on population health has led to public health bodies prioritizing the implementation of adaptive strategies to reduce the impact of climate change on health outcomes (4).

Additionally, while a common feature of national public health strategies is the involvement of stakeholders across multiple sectors, the degree to which these stakeholders may participate can vary. For example, stakeholders such as politicians and national health authorities may retain overall governance or oversight of strategy implementation at a central level, while community organizations and the public may act as cooperating partners or facilitators on strategic actions at a local level (3).

Regardless of involvement level, engagement with strategy stakeholders is essential throughout the life cycle of a national public health strategy. This includes strategy development, where clarity on the nation’s key health challenges and supporting structures is crucial to ensure successful and sustainable strategy implementation (2). Previously, inclusive population consultation methods have been used during strategy development to capture opinions and transform the public into active stakeholders in a strategy (3), however the extent to which these methods are used varies considerably worldwide.

Given the many factors which may influence population health, and the number of stakeholders who may be involved in strategy development and implementation, it is increasingly challenging to develop comprehensive strategies which positively impact national health outcomes. Furthermore, the COVID-19 pandemic has sparked a renewed political focus on public health, with a number of countries taking actions to review, strengthen or reform their national public health systems and strategies (5–7). Therefore, gaining an understanding of the issues of importance, the actions undertaken and strategy development methods used internationally may provide insights into effective methods of national public health strategy development and delivery.

In light of the timeliness and complexity of this issue, the aim of this research was to review national public health strategies in selected countries, focusing on both strategy development and strategy content (such as issues of importance and strategy implementation actions). This research was originally undertaken to inform work towards the development of a national public health strategy in Ireland. However, the findings provide insights of interest to politicians, policymakers, and individuals and organizations engaged in public health research and practice internationally.

## 2 Methods

### 2.1 Country selection and search of relevant international sources

The following 15 countries were selected for inclusion in this review: Austria, Australia, Canada, Czechia, England, Finland, France, New Zealand, Northern Ireland, Portugal, Scotland, Singapore, Spain, Sweden and Wales. These countries were selected based on a combination of factors including geographical proximity to Ireland, similar population size, organization of health services, European Union membership and or availability of documents in English. Information on national public health strategies for the selected countries was identified in May and June of 2023 and sourced primarily from government resources (websites, reports and press releases) (see Supplementary Table 1). Representatives from key national-level organizations were identified using a combination of online searches and contact points from previous public health research (8–10). Representatives were contacted for confirmation of resources and additional resources as appropriate.

### 2.2 Inclusion and exclusion criteria

To identify relevant national public health strategies inclusion and exclusion criteria were developed through scoping of the literature and discussion with international experts. The following inclusion criteria were applied. Strategies were included if they contained some, or all, of the following elements:

- A framework for improving health outcomes, including those related to the United Nations (UN) Sustainable Development Goals (SDGs), and other national priority health problems, such as non-communicable diseases (1)
- A contribution to the improvement of health outcomes by way of activities typically falling under the essential public health functions (EPHFs) (see Supplementary Table 2), as defined by the WHO (11)
- Some, or all, of the five aspects of strategy planning as outlined by Mintzberg (12): plan; ploy; pattern; position; and perspective
- Some, or all, of the ten features which facilitate strategy translation to public health practice (see Supplementary Table 3) (13).

Additionally, as all aspects of national public health strategies may not be contained within a single document and may take various forms, relevant documents included, but were not limited to:

- Broad overview strategies (for example, a strategy which includes themes or priorities and implementation actions in a single resource)
- Strategy implementation plans and or frameworks
- Strategy evaluation plans and or frameworks
- Laws, rules, regulations and policies
- Mandates.

Furthermore, when identifying relevant documents the following were considered out of scope:

- Public health service strategies, in which national health services are responsible for dealing with disease, but may not necessarily influence the major forces which cause disease (2). These strategies may focus on the delivery, transformation and or sustainability of public health services. Examples include the *NHS Long Term Plan* (United Kingdom) (14), *Healthier SG* (Singapore) (15), *HEALTH 2030: Strategic framework for healthcare development in the Czech Republic until 2030* (16) and *New Zealand Health Strategy* (17)
- Strategies published by public health agencies, faculties and or institutions, which outline organisational priorities for the delivery of public health domain functions. Examples include *Public Health Wales: Working together for a healthier Wales – Our long-term strategy 2023–2035* (18), the *UK Faculty of Public Health Strategy 2020–2025* (19) and the *Canadian Public Health Association Strategic Plan 2021–2025* (20)
- Global-focused public health strategies, such as *Public Health Wales: Our International Health Strategy 2017–2027* (21)
- Local, territorial or provincial public health strategies, such as *Healthy Canberra: ACT Preventative Health Plan 2020–2025* (22)
- Public health strategies focused on certain conditions, diseases, age groups or predeterminants, such as mental health, anti-microbial resistance, children’s health and women’s health.

Non-English documents and websites from the included countries were translated where necessary via Google Translate.

### 2.3 Data extraction and analysis of national public health strategies

Within the included documents, all relevant information was extracted via an extraction template, in May and June of 2023 (see Supplementary Table 4). This included, but was not limited to:

- Strategy

- Timelines (that a strategy applies to)
- Governance
- Scope (for example, health-specific or applying to additional areas such as the environment)
- Aims
- Themes and priorities (these are the focus areas outlined, for example, themes around empowering healthy living and addressing social determinants of health and or priorities such as equitable child and maternity health care)
- Implementation actions (for example, information around types of actions and with whom responsibility for implementation lies)
- Outcomes (for example, information around desired strategy outcomes, measurable targets and outcome measurement methods)

- Stakeholder engagement and consultation involved in strategy development and or implementation
- Economic analysis supporting strategic priorities and or themes.

The extracted information was then compared across the selected countries, and similar and contrasting elements presented descriptively.

## 3 Results

### 3.1 Strategies identified

Relevant national public health strategies were identified for ten of the 15 included countries: Australia (23), Austria (24–26), Canada (27), England (28), Finland (29, 30), Northern Ireland (31, 32), Portugal (33–35), Scotland (36, 37), Spain (38) and Sweden (39–42) (see Table 1). Current national public health strategies were not identified for Czechia, France, New Zealand, Singapore and Wales.

**Table 1:** National public health strategy documents or resources identified for ten of the 15 selected countries.

Country	Broad strategy*	Strategy implementation plan and or framework	Strategy evaluation plan and or framework	Laws, rules, regulations or policies	Strategy development or methodology
Australia	National Preventive Health Strategy: Valuing health before illness: Living well for longer (23)	N/A	N/A	N/A	N/A
Austria	Austrian Health Targets – long healthy life years for all (24) ROADMAP “Future Health Promotion”: 10 packages of measures for a health-promoting future in Austria^§^ (26)	Health promotion strategy within the framework of the Federal Target Management Agreement^§^ (25)	N/A	N/A	N/A
Canada	N/A	N/A	N/A	Creating a Healthier Canada: Making Prevention a Priority. A Declaration of Health and Health Promotion/Health Living (27)	N/A
England	N/A	N/A	Public Health Outcomes Framework (PHOF) 2019–2022 (28)	N/A	N/A
Finland	Promoting wellbeing, health and safety in 2030: A government resolution^§^ (29)	Promoting wellbeing, health and safety in 2030: Implementation plan^§^ (30)	N/A	N/A	N/A
Northern Ireland	Making Life Better – Strategic Framework for Public Health^§^ (31)	N/A	Making Life Better: Key indicators progress update 2022^§^ (32)	N/A	N/A
Portugal	National Health Plan (PNS) 2021–2030^§^ (35)	N/A	National Health Plan (PNS) 2021–2030: Projections and Prognosis^§^ (33)	N/A	National Health Plan (PNS) 2021–2030: Methodology^§^ (34)
Scotland	National Performance Framework (NPF)^‡^ (37)	N/A	N/A	Scotland’s public health priorities (36)	N/A
Spain	Public Health Strategy (ESP) 2022 (38)	N/A	N/A	N/A	N/A
Sweden	Towards good and equal health: A framework for implementing and monitoring the national public health policy^§^ (40)	Towards good and equal health: Support structure for state public health work^§^ (42)	Core indicators for follow-up of the public health policy objective^§^ (41)	Good and equal health – a developed public health policy^§^ (39)	N/A

*All broad strategies contained elements of implementation and or evaluation plans or frameworks, or strategy development. ^§^Affiliated documents and or resources (within the selected country). ^‡^While broader than public health all contents of the NPF have been included as it was identified by a key representative as a wellness framework designed to focus work in the public sector, and beyond, on shared outcomes for the population. Key: ESP: Public Health Strategy Spain 2022; NPF: National Performance Framework; N/A: Not available; PHOF; Public Health Outcomes Framework; PNS: National Health Plan Portugal (Plano Nacional de Saúde).

Multiple documents and or resources were identified for Austria (24–26), Finland (29, 30), Northern Ireland (31, 32), Portugal (33–35), Scotland (36, 37) and Sweden (39–42). These documents or resources included broad strategies; implementation plans or frameworks; evaluation plans or frameworks; laws, rules, regulations or policies; and development or methodology information. No relevant mandates were identified.

This resulted in information for an individual country identified from either:

- A single resource published by a government or a government-mandated body, such as the broad strategy identified for Australia (23)
- Multiple affiliated resources published by a government or government-mandated body, such as those identified for Finland (29, 30)
- Multiple resources published by a government and or government-mandated body, which are not clearly affiliated, such as for Scotland [the National Performance Framework (NPF) (37) and Scotland’s public health priorities (36)].

For clarity, where information was included for a country in which multiple, not clearly affiliated resources were published by a government and or government-mandated bodies, this was specified [for example, Austria (Health Targets) (24) and Austria (Roadmap (26))].

### 3.2 Strategy contents

#### 3.2.1 Timeline and aims

##### 3.2.1.1 Timeline

All national public health strategies included were in place at the time of data extraction. Of the ten countries for which national public health strategies were identified:

- Scotland (NPF and public health priorities) (36, 37), Sweden (40), Canada (27) and England (28) published national public health strategies with no timeline.
- The remaining six countries published national public health strategies with a defined timeline (dates for the beginning and end of implementation).

**Figure 1:**
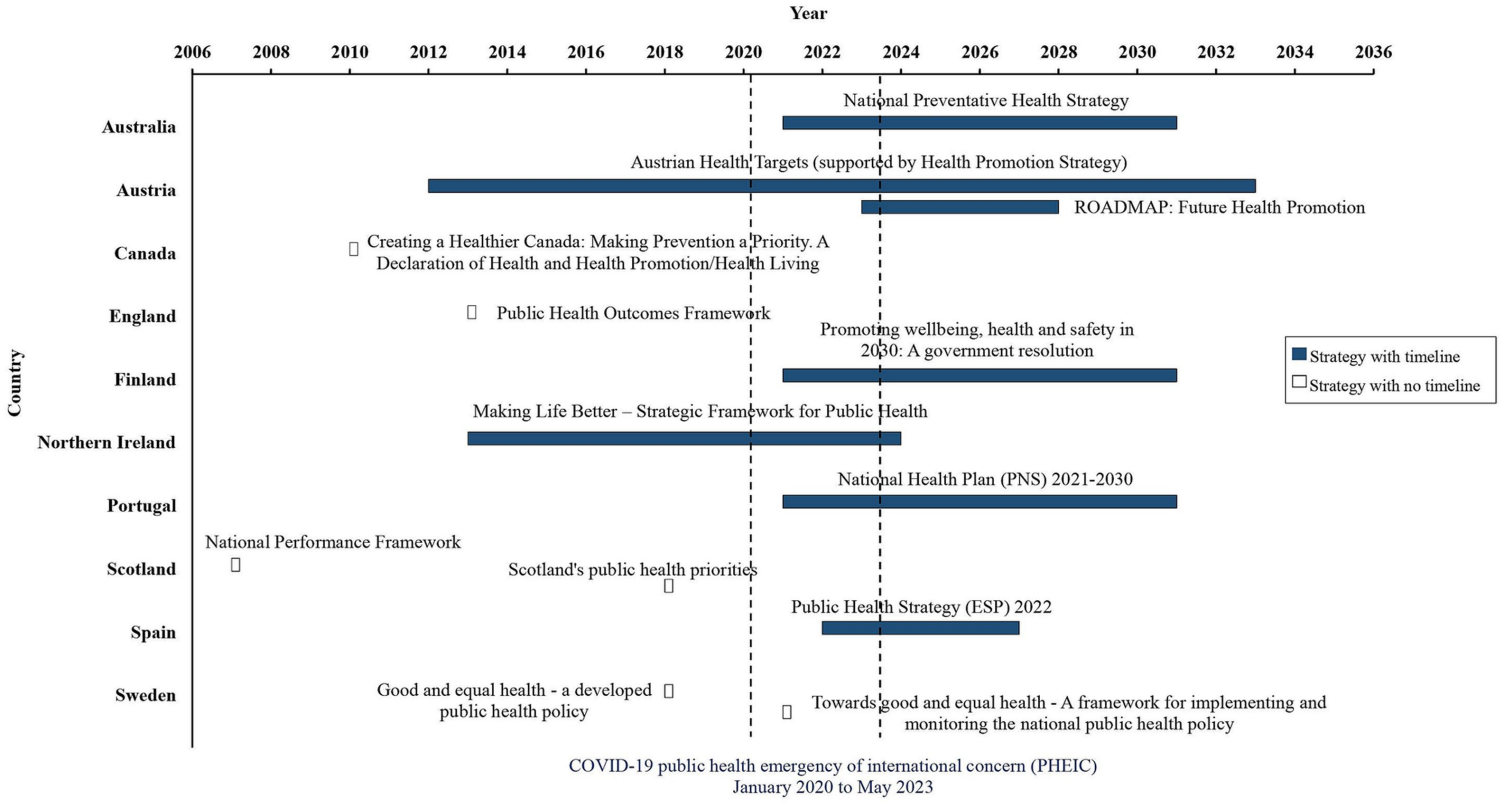
Timeline of national public health strategies identified and or confirmed by key representatives.

The earliest national public health strategy identified was the National Performance Framework (Scotland) published in 2007 (still currently in place) (37), with the most recent published by Austria [Roadmap (26)] in 2023 (see Figure 1). Within this review the COVID-19 pandemic was considered to represent the time period for which the WHO declared the pandemic a public health emergency of international concern, 30 January 2020 to 5 May 2023 (43). Six countries published a national public health strategy or strategies prior to the COVID-19 pandemic: Austria (Health Targets) (24); Canada (27); England (28); Northern Ireland (31); Scotland (36, 37); and Sweden (good and equal health policy) (39). Six countries published a national public health strategy or strategies during the COVID-19 pandemic: Australia (23); Austria (Roadmap) (26); Finland (29); Portugal (35); Spain (38); and Sweden (good and equal health framework) (40). As of July 2023, upcoming planned national public health strategy publication, was confirmed through personal communication with key representatives in England (July 2023) (44), France (Autumn 2023) (45) and New Zealand (July 2023) (46).

##### 3.2.1.2 Aims

Within the included national public health strategies the following related terms were identified: aim; vision; purpose; and goal (see Supplementary Table 5). For clarity, the term “aim” was used as a representative term within this review.

All of the included national public health strategies focused on improving health and wellbeing, with this being an underpinning general aim (see Supplementary Table 5) (23, 24, 26–29, 31, 35–40). This would be achieved through a number of identified strategic aims including:

- The elimination of health inequities and inequalities; a strategic aim for Australia (23), Austria (Health Targets) (24), Canada (27), England (28), Finland (29), Northern Ireland (31), Scotland (36, 37) and Sweden (39).
- Focusing on sustainable health; a strategic aim for Finland (29) and Portugal (35).

Only Australia outlined measurable outcomes related to overall national public health strategy aims (23). Australia specified outcomes related to life expectancy, aiming to achieve an additional two years of life lived in full health for all Australians by 2030, and at least an additional three years of life lived in full health by 2030 for:

- Australians in the two lowest Socio-Economic Index For Areas quintiles
- Australians in regional and remote areas
- Aboriginal and Torres Strait Islander people.

Australia also outlined further measurable outcomes related to early life (for example, the proportion of Aboriginal and Torres Strait Islander babies with a healthy birthweight will increase to at least 91% by 2031) and investment in health prevention (to reach 5% of total health expenditure across Commonwealth, state and territory governments by 2030) (23).

#### 3.2.2 Scope and alignment with international aims

All of the included national public health strategies demonstrated a multisectoral approach, with scope extending beyond the health domain (see Supplementary Table 6 and Section 3.2.3 Themes or priorities). The UN SDGs (47) and WHO Targets were frequently identified as reference points with which national public health strategies were aligned.

Two countries directly aligned multisectoral strategy aims and or themes or priorities with the UN SDGs:

- Australia (23) aligned their overall strategy aims with SDG 3: “Good Health and Well-Being” and SDG 5: “Gender Equality”
- Spain (38) aligned the indicators associated with their strategic actions with SDG 2 “Zero Hunger”; SDG 3: “Good Health and Well-Being”; SDG 5: “Gender Equality”; SDG 6:“Clean Water and Sanitation”; SDG 11: “Sustainable Cities and Communities”; SDG 12: “Responsible Consumption and Production” and SDG 16: “Peace, Justice and Strong Institutions.”

Finland (29), Scotland (NPF) (37) and Sweden (40) also stated that their strategies reflected and or broadly aligned with the UN SDGs, however direct alignment was not identified. Australia (23) aligned their overall strategy aims with a number of WHO Targets including the WHO ‘Healthier Populations’ triple billion goal, WHO ‘Universal Health Coverage’ triple billion goal and WHO Global nutrition targets 2025.

Alignment with a whole-of-government approach was outlined by Australia (23) and Portugal (35), and a whole system approach was outlined by Northern Ireland (31). Collaboration with multiple government departments, ministries, and or agencies was outlined by Australia (23), Finland (29), Northern Ireland (31), Scotland (36, 37) and Spain (38). Collaboration with the private sector; private and public sector; or private, public and third sectors was outlined or emphasized by Australia (23), Sweden (40) and Scotland (public health priorities) (36) respectively. Working with alternative sectors to enhance economic growth was also outlined within the strategy scope of Canada (27), Finland (29) and Scotland (NPF) (37).

#### 3.2.3 Themes or priorities

Within the included national public health strategies the following related terms were identified: themes; priorities; target areas; goals; focus areas; strategic action lines; and core responsibilities (see Supplementary Table 7). For clarity, the term ‘themes or priorities’ was used as a representative term within this review.

Themes or priorities were identified in the majority of included national public health strategies. All of the countries included a theme or priority around promoting healthy living or behaviors, while the majority of countries included a theme or priority around the strengthening of public health; equity or equality; and working together or collaborating (see Table 2). Following this, themes or priorities were also identified around climate change and the environment; early life, children and youth; working life; and empowering citizens. Themes or priorities around mental health; preparing for or adapting to threats; disease prevention; and maintaining the health status were less frequently identified.

**Table 2:** Occurrence of themes or priorities identified in the national public health strategies of selected countries.

Theme or priority domain	Australia	Austria	Canada	England	Finland	Northern Ireland	Portugal	Scotland	Spain	Sweden	Number of countries
Promoting healthy living or behaviors*	✓	✓	✓	✓	✓	✓	✓	✓	✓	✓	**10/10**
Strengthening of public health	✓	✓	X	X	✓	✓	✓	X	✓	X	**6/10**
Equity or equality^†^	✓	✓	X	X	X	X	✓	✓	✓	✓	**6/10**
Working together or collaborating	✓	✓	✓	X	✓	✓	X	✓	X	X	**6/10**
Climate change or environment	X	✓	X	X	✓	✓	✓	✓	X	X	**5/10**
Early life, children and youth	X	✓	X	X	X	✓	X	✓	X	✓	**4/10**
Working life	X	✓	X	X	X	✓	X	✓	X	✓	**4/10**
Empowering citizens	✓	✓	X	X	X	✓	X	✓	X	X	**4/10**
Mental health	X	✓	X	X	X	X	X	✓	X	X	**2/10**
Preparing for, or adapting to, threats	✓	X	X	X	X	X	X	X	✓	X	**2/10**
Disease prevention	X	X	✓	X	X	X	X	X	X	X	**1/10**
Maintaining the health status	X	X	X	X	X	X	✓	X	X	X	**1/10**

*Includes themes or priorities around smoking, drugs, nutrition and physical activity. ^†^Includes themes or priorities around equity in income and housing.

##### 3.2.3.1 Promoting healthy living or behaviors

Promoting healthy living or behaviors was identified as a theme or priority within the national public health strategies of all 10 countries (see Supplementary Table 7). Focus areas included the promotion of healthy eating and or a sustainable food system, prioritized by Austria (24, 26) and Scotland (36, 37); the promotion of healthy and safe exercise, prioritized by Austria (Health Targets) and Scotland (36, 37); and the reduction of tobacco, alcohol and or drug use, prioritized by Scotland (public health priorities) (36).

##### 3.2.3.2 The strengthening of public health

The strengthening of public health was identified as a theme or priority within the national public health strategies of six countries [Australia (23), Austria (24, 26), Finland (29), Northern Ireland (31), Portugal (35) and Spain (38)] (see Supplementary Table 7). Examples included the delivery of high quality, safe and/or affordable health and care services as a priority for Austria (Health Targets); enabling the workforce in terms of skills and resources in Australia (23) and Austria (Roadmap); and increasing digital capabilities, such as data and surveillance capabilities, in Australia (23).

##### 3.2.3.3 Equity or equality

Ensuring health equity and or reducing health inequalities was identified as a theme or priority within the national public health strategies of six countries [Australia (23), Austria (Health Targets) (24), Portugal (35), Scotland (36, 37), Spain (38), and Sweden (40)] (see Supplementary Table 7). More specifically, Scotland (NPF) (37) prioritized equality in relation to opportunities, wealth, power and human rights, while Sweden prioritized equity in regards to medical services and equality in relation to the distribution of income, financial resources and housing (40).

##### 3.2.3.4 Working together or collaborating

Working together or collaborating was identified as a theme or priority within the national public health strategies of six countries [Australia (23), Austria (24, 26) Canada (27), Finland (29), Northern Ireland (31) and Scotland (NPF) (37)] (see Supplementary Table 7). Both Austria (Health Targets) (24) and Canada (27) outlined the importance of collaboration in regards to health promotion activities. Working together with stakeholders was also identified, with collaboration at community (Australia and Austria) (23, 24, 26), international (Scotland – NPF) (37) and all levels [Finland (29) and Northern Ireland (31)] identified as priorities.

##### 3.2.3.5 Climate change or environment

Minimizing the impact of climate change and or ensuring a healthy environment was identified as a theme or priority within the national public health strategies of five countries [Austria (24, 26), Finland (29), Northern Ireland (31), Portugal (35) and Scotland (36, 37)] (see Supplementary Table 7). Austria (Health Targets) (24) focused on specific components of the environment, outlining a focus on air, water, soil and habitat preservation.

##### 3.2.3.6 Early life, children and youth

Providing children with the best start in life was identified as a theme or priority within the national public health strategies of Austria (24, 26), Northern Ireland (31), Scotland (36, 37) and Sweden (40) (see Supplementary Table 7). More specifically Austria (Roadmap) (26) and Sweden (40) outlined the importance of a health-promoting learning environment and equal education system, while Sweden also linked equal maternal and child health care within their strategy (40).

##### 3.2.3.7 Working life

Working life was identified as a theme or priority within the national public health strategies of Austria (Roadmap) (26), Northern Ireland (31), Scotland (NPF) (37) and Sweden (40) (see Supplementary Table 7). Austria (Roadmap) (26), Scotland (NPF) (37) and Sweden (40) focused on working conditions and the promotion of health promoting, sustainable and innovative work practices, while Northern Ireland (31) focused on the preparation of young people for their transition into adult working life.

##### 3.2.3.8 Empowering citizens

Empowering citizens was identified as a theme or priority within the national public health strategies of Australia (23), Austria (24, 26), Northern Ireland (31) and Scotland (NPF) (37) (see Supplementary Table 7). Australia (23), Northern Ireland (31) and Scotland (NPF) (37) broadly identified empowering and supporting communities as a priority, while Austria (24, 26) focused on strengthening the health literacy of the population. Scotland (NPF) (37) also prioritized empowering citizens through providing education and ensuring cultures are expressed and enjoyed.

##### 3.2.3.9 Mental health

The promotion of improved mental health, well-being and or psychosocial health was identified as a theme or priority within the national public health strategies of Austria (24, 26) and Scotland (public health priorities) (36) (see Supplementary Table 7). The elimination of mental health taboos was emphasized by Austria (Roadmap) (26).

##### 3.2.3.10 Preparing for, or adapting to, threats

Preparing for or adapting to threats was identified as a theme or priority within the national public health strategies of Australia (23) and Spain (38) (see Supplementary Table 7).

##### 3.2.3.11 Maintaining the health status

Maintaining the health status was only specifically identified as a theme or priority within Portugal’s national public health strategy (35) (see Supplementary Table 7). Portugal outlined ‘Keeping health problems under control’ as a theme of the National Health Plan (PNS), along with the associated strategic objectives of ensuring quality sexual and or reproductive, maternal and child health, surveillance and care; maintaining immunization coverage; and keeping water-borne health problems under control (35).

##### 3.2.3.12 Disease prevention

Disease prevention was only specifically identified as a theme or priority within Canada’s national public health strategy (27). Canada (27) outlined disease prevention as a priority, a hallmark of a quality health system and the first step in management.

#### 3.2.4 Governance

An outline of key governance structures was included in the national public health strategies of seven countries: Australia (23), Austria (Health Targets) (24), Finland (29), Northern Ireland (31), Portugal (35), Scotland (NPF) (37) and Spain (38) (see Supplementary Table 8). A high level description of a governance mechanism at federal level was provided by Australia (23) and Scotland (NPF) (37), while in contrast Northern Ireland provided an in-depth description of key governance structures, from local level (community planning and local strategic partnerships) to regional (Regional Project Board) and national level (Ministerial Committee for Public Health) (31).

Specifically, governance by their respective Minister or Ministry of Health was outlined by Austria (Health Targets) (24), Northern Ireland (31), Portugal (35) and Spain (38) with strategy coordination, implementation and or support outlined as a ministerial function. Portugal also identified the Directorate-General for Health (DGS) as responsible for coordinating strategy planning and the execution of PNS 2021–2030 (35). Specific boards or institutes tasked with the monitoring of strategies were identified for Finland (Public Health Advisory Board) (29, 30), Portugal (National Health Institute Dr. Ricardo Jorge) (35) and Spain (State Centre for Public Health) (38), while Northern Ireland outlined monitoring would occur from local to Ministerial level, with reports produced at each level (31).

Information around national public health strategy governance was not identified for Austria (Roadmap) (26), Canada (27), England (28) and Scotland (public health priorities) (36). Sweden outlined that shared responsibility and joint inter-agency dialogue occurs, however this is not formalized (40).

#### 3.2.5 Implementation

Details on implementation were outlined to varying extents in all of the included national public health strategies. Specifically, these details included descriptions of the planned strategy implementation actions, the stakeholders responsible for leading implementation and or distinct aspects of implementation, and key performance indicators for monitoring and evaluating strategy implementation.

##### 3.2.5.1 Actions

Within the included national public health strategies the following related terms were identified: actions; measures; activities; approaches; objectives; steps; procedures and commitments (see Supplementary Table 9). For clarity, the term ‘actions’ was used as a representative term within this review.

For the majority of countries with included national public health strategies, implementation actions, either planned or in progress, were outlined. The contents of the actions were related to the themes or priorities of the strategy, and the level of detail with which these actions was described varied between countries. Summaries and or samples of actions are outlined in Supplementary Table 9.

Implementation plans containing detailed actions were provided for Finland (30), Northern Ireland (31), Scotland (public health priorities) (36) and Spain (38). A distinct implementation plan document was published to accompany the Finnish national public health strategy (30). This implementation plan outlined sub-goals for each of the four focal points listed in Section 3.2.3, Themes or priorities, along with their associated actions, timelines, coordinating bodies or leads, and cooperating partners. Similarly detailed actions aligned with strategic themes or priorities were included within the national public health strategies of Northern Ireland (31) and Spain (38). For Northern Ireland (31), other key strategies and or strategic programs linked to implementation were also identified for each theme. For example, supporting policies and initiatives for ‘Theme 1: Giving every child the best start’ included the School Improvement policy – “Every School a Good School” (48), the Families Matter strategy (49), and the Community Family Support Programme (50).

In contrast to the detailed implementation plans identified, high-level strategic actions were observed for Australia (23), Austria (Health Targets) (24), Portugal (35) and Sweden (40). For Australia (23) Portugal (35) and Sweden (40) the principles to be adopted to support implementation were outlined, but details of how the priorities or strategic actions should be achieved were not described. However, while the Swedish national public health strategy itself did not contain detailed actions (40), an affiliated document, in the form of a year one overview report of implementation, included summary information of actions in progress and planned by the Public Health Agency (42). For Australia, a ‘Blueprint for Action’, outlining implementation details for the national public health strategy, was reported to be in development (not published as of 2nd October 2024) (51).

##### 3.2.5.2 Leads

Leads, key partners, authorities and or institutions responsible for the implementation of actions were specifically outlined in the national public health strategies of Austria (Health Targets) (24), Finland (30), Northern Ireland (31), Spain (38) and Sweden (40) (see Supplementary Table 9):

- Austria (Health Targets) (24) outlined the institutions responsible for implementing measures to achieve the desired outcomes. Examples included: the Federal Ministry for Social Affairs, Health, Care and Consumer Protection; Federal Ministry of Education, Science and Research and the Healthy Austria Fund.
- Finland (30) and Spain (38) outlined coordinating bodies or leads, and or cooperation partners associated with their strategic actions. Examples for Finland included: the Ministry of Economic Affairs and Employment; the Department of Health and Welfare and the Institute of Occupational Health; and the Ministry of Health for Spain.
- Northern Ireland outlined key partners involved in the implementation of actions and commitments (31). Examples included: the Department of Health, Social Services and Public Safety, Children and Young People’s Strategic Partnership and the Social Security Agency.
- Sweden outlined authorities responsible for important issues in each target area and also a number of authorities relevant to all target areas (40). Examples included: the Ombudsman for Children, the Non-Discrimination Ombudsman and the Swedish Gender Equality Agency.

Scotland (public health priorities) did not explicitly state leads associated with each strategic action but sporadically mentioned collaborating partners, and or agencies that would be supported in action implementation (36).

##### 3.2.5.3 Key performance indicators

Key performance indicators (KPI) associated with strategy themes and or implementation actions were outlined for seven countries [England (28), Finland (30), Northern Ireland (31), Portugal (35), Scotland (NPF) (37), Spain (38) and Sweden (40)] (see Supplementary Table 9). For the majority of countries, KPIs were linked to strategy themes or priorities, and the number of indicators outlined varied considerably between countries. For example, Finland’s national public health strategy is linked with 3,500 KPIs on health, welfare and the functioning of the health service system (52).

England (28), Northern Ireland (31), Spain (38) and Sweden (40) also outlined overarching KPIs. Life expectancy (28, 31, 38, 40) and healthy life expectancy or years (28, 31, 38) were frequently-identified overarching KPIs, with further examples including disability-free life expectancy (31), deaths by cause of death (38) and self-assessed general state of health (40). Austria (health promotion strategy) (25) and Portugal (35) also outlined targets associated with KPIs, such as a maternal mortality rate equal to or less than 7.1 per 100,000 live births, in the three-year period of 2028–2030 (Portugal) (35). Australia (23) and Austria (Health Targets) (24) outlined a number of desired policy achievements or outcomes, however, no associated KPIs were outlined.

#### 3.2.6 Strategy development

With the exception of Canada, information on how the strategies were developed was reported for all of the included national public health strategies. However, the extent of the strategy development information reported varied greatly between countries.

Detailed descriptions of strategy development processes were reported for Austria (24, 26), Portugal (35) and Sweden (40). For Portugal, a discrete methodology document was published in relation to their national public health strategy (34, 35). Details of distinct stages in the strategy development process were reported for the following countries:

- Identification of priorities [Austria (24, 26), Portugal (34), Spain (38) and Sweden (40)]
- Drafting of the strategy [Austria (24, 26), Portugal (34) and Sweden (40)]
- Development of recommendations for implementation [Austria (Roadmap) (26) and Portugal (34)]
- Identification and selection of indicators for monitoring and evaluation [Spain (38) and Sweden (40)]
- Development of a monitoring and evaluation plan [Portugal (34)]
- Development of a communication plan to support the strategy [Portugal (34)].

Timelines for strategy development were reported for Austria (24, 26), Northern Ireland (31) and Portugal (34) with timelines ranging from approximately 12 months [Austria (Health Targets) (24)] to three years (Portugal) (34).

Overall, national public health strategies were observed to have been developed using evidence-based, collaborative approaches involving stakeholders across multiple sectors, led by a relevant government or government-mandated body with responsibility for public health. Common strategy development methods observed may be broadly categorized as evidence review and stakeholder engagement. These methods were used to draw on information, expertise and experiences from across a range of relevant sectors, in keeping with the multisectoral nature of national public health strategies.

##### 3.2.6.1 Evidence review

‘Evidence review’ is used in the current review to broadly describe any methods of finding and summarizing published research and or other literature. Evidence review was used to inform the development of national public health strategies for seven countries [Australia (23), Austria (Health Targets) (24), Northern Ireland (31), Portugal (34), Scotland (public health priorities) (36), Spain (38) and Sweden (40)]. Evidence review methods were not specified in detail for any country. Where the sources of evidence used to inform strategy development were stated, they included:

- relevant international, national and regional strategies, frameworks and action plans [Australia (23), Austria (Health Targets) (24), Northern Ireland (31), Portugal (34), Scotland (36, 37), Spain (38) and Sweden (40)]
- national and international scientific and grey literature [Australia (23), Northern Ireland (31), Scotland (public health priorities) (36) and Spain (38)]
- national legislation [Spain (38) and Sweden (40)].

Data obtained from national and or international health information systems were also used to inform strategy development in Spain (38) and Sweden (40). In Spain (38), these data informed priorities for action in public health based on factors such as health-related impacts, and economic and societal costs. In Australia, Australian Government Department of Health and Aged Care (23) lessons learned from past disease prevention activities were also used to inform strategy development.

The stated purposes of reviewing evidence included informing the development of the themes or priorities of the strategy in three countries: Scotland (public health priorities) (36), Spain (38) and Sweden (40); identifying impactful public health interventions in Australia (23), and ensuring that the strategy aligned with existing priorities and actions in two countries: Australia (23) and Scotland (public health priorities) (36). In Sweden (40), this included alignment with national budgetary priorities, specifically national expenditure targets for areas that may support the realization of public health policy goals. In Portugal (34), a review of evidence was used to compile an initial comprehensive matrix of intervention strategies, which was then used as the basis for intervention strategy selection through an extensive stakeholder engagement process.

The timing of the evidence review within the context of overall strategy development was only reported for Austria (Health Targets) (24), where it took place throughout the main period of strategy development work, in tandem with targeted stakeholder engagement.

##### 3.2.6.2 Stakeholder engagement

Engagement with stakeholders across multiple sectors was a common feature in the development of national public health strategies in the selected countries. The methods used may be broadly considered as public consultation or targeted consultation methods (see Supplementary Table 10).

Public consultation was used to inform the development of national public health strategies for Australia (23), Austria (24, 26), Northern Ireland (31) and Portugal (34). Methods used included surveys [Australia (23) and Austria (Roadmap) (26)]; online public consultations [Australia (23), Austria (Health Targets) (24) and Portugal (34)]; citizen councils [Austria (Roadmap) (26)] and regional engagement events [Scotland (public health priorities) (36)].

Targeted consultation methods were used to inform the development of national public health strategies in eight countries. In all cases, targeted consultation methods were used to engage with stakeholders with expertise and or experience relevant to the development and or implementation of the national public health strategy. These stakeholders included experts in public health and strategy, representatives from national, regional and local government, representatives from relevant government-mandated entities, trade union representatives, and stakeholders representing relevant voluntary organizations. Targeted consultation was also used as a supplement to public consultation in Austria (Roadmap) (26). In that case, focus groups were conducted in order to engage with specific groups of people who may have experienced barriers to participating in other public consultation methods, such as young people, older people, people at risk of poverty, people with migratory backgrounds (people who had either migrated to the country or at least one of their parents previously entered the country as a migrant), and people with disabilities and their carers.

Australia (23), Austria (Health Targets) (24) and Portugal (34) established groups comprising experts and or representatives from relevant sectors and organizations to contribute throughout the strategy development process. In Australia (23), an expert steering committee guided the overall development of the strategy. In Austria (Health Targets) (24) a group consisting of 40 stakeholders from various political and social sectors contributed through workshops in which they developed a proposal for health targets for the Federal Health Commission. These stakeholders also provided feedback on the health targets to their organizations in order to ensure broad acceptance of the goals across relevant sectors. In Portugal (34), the national health strategy was developed using a co-creation approach. This involved collaborative working throughout the development process with an Advisory Board, comprising expert stakeholders, and a Monitoring Commission, made up of representatives from a range of relevant sectors. In addition to the involvement of the Advisory Board and Monitoring Commission, other distinct targeted consultation methods, such as multisectoral seminars and a mixed-methods health goals setting exercise with separate stakeholder groups, were also conducted during the strategy development process.

Other stakeholder engagement methods included hosting workshops to provide opportunities for specific stakeholder groups to contribute to the strategy [Finland (29) and Northern Ireland (31)]; participating in strategic dialogues with representatives from government and other relevant authorities (Sweden) (40); and presenting and discussing the strategy at national health conferences (Austria) (24, 26).

#### 3.2.7 Supporting economic analysis

None of the selected countries included economic analysis to support their national public health strategy(ies).

## 4 Discussion

As outlined by the WHO, national public health strategies “play an essential role in defining a country’s vision, policy directions and strategies for ensuring the health of its population” (1). Complex in nature, national public health strategies need to address national health policies, and align with global health initiatives, while also considering national democratic structures in place and political will at any given time (3). An understanding of how countries develop national public health strategies worldwide, along with understanding the health issues of importance to them and how these may be improved upon, may provide insights into effective methods of national public health strategy development and delivery. An international review of national public health strategies was therefore undertaken, with national public health strategies identified for 10 of 15 countries, selected based on a combination of factors including geographical proximity to Ireland and population size.

The review raised several questions about evaluating the impact of national public health strategies. Key performance indicators (KPI) were linked to strategy themes or priorities in the majority of countries, but the number of indicators outlined varied considerably between countries and two countries had no associated KPIs. Life expectancy, healthy life expectancy or years, disability-free life expectancy, deaths by cause of death, self-assessed general state of health and maternal mortality rate were among the KPIs used in the public health strategies reviewed. The identification of internationally agreed indicators to measure the implementation of public health strategies and assessing the impact on health outcomes of their targeted populations would be valuable. However, only Australia cited outcomes that they wished their strategy to impact. Even then it is difficult to state with certainty that changes in a high level outcome (in this case relating to elements of life expectancy) are attributable to inputs such as a strategy. This is clearly an important area for future health services research. There is an even more fundamental question as to whether the presence or absence of a strategy (and there were four countries which appeared not to have a current one) is associated with overall better, worse or equal health outcomes. This is yet another area which requires further research.

The questions raised by us are: (a) what international core set of themes or priorities should be included in the development of a national public health strategy; (b) what indicators should be used to measure the implementation of a strategy and impact on health outcomes; (c) what is the optimal combination of prescriptive or interpretative approaches depending on national contexts; (d) which elements of governance are critical to successful implementation including the balance of evidence gathering versus stakeholder engagement.

On core priorities, several key aims were identified across the included national public health strategies. These relate to the United Nation’s Sustainable Development Goals (47) and included elimination of health inequities and inequalities; keeping people safe and or monitoring emerging threats; and focusing on sustainable health. These contributed to an over-arching aim identified in all of the included national public health strategies, that is, to improve health and wellbeing. There is good evidence and previous work to guide appropriate responses to these aims (53–55).

In terms of specific priorities, it is clear that many countries aligned around promoting healthy living – the importance of which was demonstrated by the impact of the COVID-19 pandemic on vulnerable populations (55–58). Almost half of the national public health strategies included within this review were published during the COVID-19 pandemic and the experience of the pandemic period may have contributed to the prioritization of promoting healthy living or behaviors. Furthermore, during priority setting, health stakeholders try to preempt foreseeable health problems and mitigate their impact on health outcomes. It is perhaps surprising that preventing ill health was explicitly stated as a priority by only one country. While this might suggest a disconnect with health improving behaviors, it is also important to consider that countries may be addressing preventing ill health within health promotion strategies, rather than disease prevention. Further priorities found in several strategies include actions on climate change and health; actions requiring cross government and cross sector engagement; and strengthening the public health system and workforce. Given the critical role of public health during the COVID-19 pandemic, it is unsurprising that the strengthening of public health was regularly identified as a national public health strategy priority. This is well supported throughout the literature, where improving health workforce resilience and preparedness (56), improving public health training (57) and improving public health infrastructure are all viewed as opportunities for improvement in the post-pandemic period (58). Furthermore, tackling climate change has been described as the ‘greatest global health opportunity’ of the 21st century, and many of the mitigation and adaptation measures for climate change may also benefit population health (59). Additionally, while the wider determinants of health were not specifically identified as strategy priorities or themes, they were included as underpinning considerations throughout all of the national public health strategies. This is supported by the WHO, who have highlighted that the social determinants of health account for 30 to 55% of health outcomes, and that the contribution of sectors outside of health exceeds the contribution of the health sector to population health outcomes (60).

However, while a number of common themes or priorities were identified across the included national public health strategies, political context and health structures vary by country and this can influence the emphasis which is placed on any given priority within a strategy (61). For example, a recent overview of health policy responses to the COVID-19 pandemic found that political leadership, health system organization, and financing impacted a country’s pandemic responses.

The question as to the appropriateness of prescriptive or interpretive approaches bears scrutiny. The answer may depend to some extent on the pre-existing infrastructure of the country, and its familiarity with centralized approaches to accountability. So here, approaches adapted to countries’ cultures and capabilities may be in play. Prescriptive approaches included detailed implementation plans with specific actions and KPIs, such as those described for Finland (30) and Spain (38). More interpretive approaches to implementation were observed for Australia (23), Portugal (35) and Sweden (40). In these countries’ strategies, priorities or high-level strategic actions were outlined, along with principles to support their implementation, rather than specific actions detailing how these priorities were to be achieved. This overarching interpretive approach is necessary due to the decentralized decision-making arrangements across states and territories in Australia, and at local and regional levels in Sweden. In Portugal, sector-specific implementation strategies are planned to be developed at national and regional levels, for the health sector and for other sectors (35). Regardless of the approach used, it should be noted that the inclusion of implementation strategies or frameworks within public health strategies, does not necessarily reflect the actual implementation of the associated strategies. Effective implementation of national public health strategies depends on a number of factors, including but not limited to the population’s willingness to participate and collaborate with local authorities; political and health system structures; contextual factors such as policy relevant to the strategy; and the skills, resources and opportunities available to any nation (62). For example, across a federation like Canada, significant challenges to achieving consistent, coordinated approaches to public health may occur, due to factors such as decentralized decision-making and fragmented systems for the collection and sharing of data (63). These data collection and sharing challenges may influence or limit the selection of KPIs monitored during strategy implementation, whereas a country with advanced national health information systems, such as Finland (29), utilizes a comprehensive number of KPIs. In regards to effective public health strategy implementation, Khorram-Manesh et al. (62) identified that educational initiatives and appropriate communication to the community in a timely manner are key for increasing a population’s willingness to participate and collaborate in a strategy. This is further expanded by Bialek et al. (64) who outline six steps to engaging a community in developing and implementing a collaborative strategy: 1. Establish the urgency and commitment to collaborate on selected health issues; 2. Introduce the new concepts, techniques, and tools for managing a community strategy; 3. Engage the coalition in co-creating a strategy map; 4. Distribute the work of strategy execution; 5. Adopt shared strategy measures and a shared measurement system; and 6. Harness, align, and monitor the actions.

Additionally, strict adherence to a public health strategy during implementation is not necessarily required for effective implementation, and adaptions may enhance implementation, for example modifying programmes to meet local needs.

The role of governance in successful development and particularly implementation of national health strategies is to some extent related to the way countries best operate in getting action on new national initiatives. However, there is also universal evidence on good governance, vital to effective implementation, founded on principles such as responsiveness, competence, transparency and accountability (65). A number of the included national public health strategies relied upon existing, well-established governance structures to oversee strategy implementation. For example, in Sweden (40), the strategy was built upon existing structures, divisions of responsibilities, and cooperative ways of working between government agencies that are set out in national legislation. A number of countries specified that strategy governance came under the remit of the Minister or Ministry of Health, or equivalent (24, 31, 35, 38). Existing boards or institutes with responsibilities relating to public health were tasked with strategy oversight in Finland (29, 30), Portugal (35) and Spain (38). In Australia, a need to enhance existing governance structures was identified as part of the national public health strategy, and this enhancement was proposed to be achieved through establishing an independent, expert-led mechanism along with a mechanism within government across relevant portfolios (23). Of the selected countries, the most detailed description of governance structures was provided for Northern Ireland (31), where new governance structures were established to oversee the national public health strategy at local, regional and national levels. Overall, in the majority of the included national public health strategies, it was apparent that governance structures, and where enhancements or new structures may be required, were considered as part of the strategy development process.

By their nature, national public health strategies involve multisectoral stakeholder engagement (2), and the strategy development processes observed in the selected countries tended to reflect this. In general, the included national public health strategies were developed using evidence-based, collaborative approaches involving stakeholders across multiple sectors, led by a relevant government or government-mandated body with responsibility for public health. However, while evidence review of published research, literature and other national public health strategies was conducted by seven countries, there was little detail of what this entailed. Notably, the included national public health strategies omitted economic analysis to support their development and or implementation plans. Cost–benefit analyses have become increasingly important for government departments and policy makers to gain political support for funding, as well as to assess the return-on-investment of public health interventions in addition to the benefits of a healthy population.

However, as expected, wider stakeholder engagement, such as public consultations and the formation of intersectoral partnerships, was a feature of strategy development in the majority of the included national public health strategies (3). A range of stakeholder groups were consulted including young people, older people, people at risk of poverty, people with migratory backgrounds, and people with disabilities and their carers.

The use of a variety of methods to include the public in strategy development is in keeping with the increasing emphasis on public and patient involvement (PPI) across health research and policy development in general (66, 67). PPI aims to support the development of policies or strategies that are responsive, feasible and supported by all relevant stakeholders through including people’s voices in decision-making, and achieving mutual understanding and increased trust (66, 67). Although there is little evidence currently available on the extent to which PPI has influenced public health policymaking, research has identified a variety of PPI methods that may be appropriate for use when developing health policy (66), factors that may influence the effectiveness of PPI in the health context (66, 67), and “essential” and “desirable” principles for optimizing PPI (68).

Stakeholders with expertise and experience in relevant fields contributed to strategy development in all included countries. Methods used to engage with expert stakeholders varied in the level of participation involved, ranging from stand-alone workshops or opportunities to submit contributions, as seen in Finland (29), to more participatory approaches where expert groups were convened for the purposes of contributing throughout the strategy development process, as seen in Australia (23), Austria (24, 26) and Portugal (35). In Finland, stakeholder engagement allowed for direct input on the strategy implementation plan, with stakeholders developing specific actions, and identifying the partners associated with these actions (29). These participatory approaches draw on design theory and practice, with participatory design and co-design becoming increasingly influential in the design of public services, including health and social services, and public policy (69–71). The proposed benefits of co-design are similar to that of PPI – improved idea generation, shared understanding among stakeholders, development of more feasible and acceptable solutions. However, shared challenges also exist, such as the time-consuming nature of implementing these approaches (69). Although somewhat delayed by the COVID-19 pandemic, the co-creation approach used to develop the Portuguese national public health strategy took three years to complete (35), the longest duration of the strategy development processes observed in this review. It is possible at least to measure the success and degree of public engagement in government strategies through well designed feedback and it is an approach that would have been valuable to see in the strategies reviewed.

### 4.1 Limitations

While this review presents a comprehensive descriptive analysis of national public health strategies, with strategies identified for 10 countries, there are notable limitations. Firstly, this review is a purely descriptive analysis of the included national public health strategies, with no strategy evaluation included. This is primarily the case as national public health strategies prioritize and consider health issues at a national level, which may vary from country to country. This, along with considerable differences in health systems and health service delivery internationally, limits the ability to evaluate which strategy aspects may be successful in different countries. Strategies for improving public health are complex and often situation-dependent, meaning implementation strategies used in one country would not always be possible in another (2). Following this, the countries selected for inclusion may not entirely represent the scope of national public health strategies published internationally. Countries included were selected based on factors such as geographical proximity to Ireland, a similar population size, and a similar organization of health services, in line with the original purpose of the research. Therefore, this review is not an exhaustive review of national public health strategies internationally. Additionally, national public health strategies were only identified for 10 of the 15 countries selected for inclusion, with strategies not identified for Czechia, France, New Zealand, Singapore and Wales. While key representatives for France, New Zealand, Singapore and Wales confirmed the absence of national public health strategies, contact could not be made with Czechia. This may have resulted in the omission of a national public health strategy. Similarly, while key representatives for all selected countries were contacted to confirm resources and or provide additional resources of relevance, contact could not be reached with Canada, Czechia, Finland and Spain. While national public health strategies were identified within the document search for Canada (27) Finland (29, 30) and Spain (38) these were not confirmed by key representatives.

Upcoming national public health strategy publications (at the time of data extraction) were also confirmed by key representatives from England, France and New Zealand. However, due to time constraints these were not included within the current review. Of those confirmed for upcoming publication, the New Zealand Health Strategy 2023 was published on 12th July 2023 as part of a suite of strategies (72), and the United Kingdom Health Security Agency (UKHSA) strategic plan was published on 25th July 2023 (73). While outlining aims similar to those identified within the current review (achieving health equity and improving health outcomes), the New Zealand Health Strategy 2023 outlines six strategic priorities which are mainly aligned to health service issues (such as valuing our workforce and flexible, appropriate care) (72). Thus this strategy is out of scope for the current review. The UKHSA strategic plan outlines the government-mandated agency’s role in preparing for and responding to health threats, mainly in England. Strategy goals are related to preparing for and preventing future health threats; saving lives through priorities such as reducing the impact of infectious disease and antimicrobial resistance; and developing the UK’s health security capacity (73). These are broadly in line with strategy aims identified within this review around protection from, and the monitoring of, emerging threats (73). However, this strategy focuses predominantly on health protection and therefore is deemed out of scope for the current review also. The upcoming French strategy was not published as of 22nd January 2024, however a public consultation on a draft of the French National Health Strategy 2023–2033 closed on 2nd of October 2023 (74). Once finalized, this overarching strategy will provide the framework for national health policy in France over the next 10 years, with public health to the forefront across all such policies.

## 5 Actionable recommendations

The current review of national public health strategies in selected countries provides insights into what countries identified as issues of importance, the actions undertaken, and strategy development methods used internationally. These insights may aid in the development of an evidence-based national public health strategy. There are a number of notable recommendations:

- National public health strategies are essential to define a country’s vision, policy direction and approaches to improve the health and wellbeing of its population. They provide roadmaps to prevent disease, reduce health inequalities and respond to emerging health threats.
- National public health strategies need to include a whole of government approach to address the determinants for cross-sectoral issues such as housing, air quality and access to health care. The WHO highlights that the social determinants of health account for 30 to 55% of health outcomes, and that the contribution of sectors outside of health exceeds the contribution of the health sector to population health outcomes.
- All countries reviewed identified the promotion of healthy living or behaviors as a major theme and strengthening public health as a priority in their strategies. Future attention as to whether observed health outcomes are attributable to a national strategy would be valuable, although challenging to measure. More fundamental would be an understanding of the health and economic impacts of having any public health strategy.
- The increasing impact of air, water, soil pollution and climate change on population health needs to be addressed in a national public health strategy, including alignment with global health initiatives such as the UN SDGs and WHO’s Targets.
- Identification of country-specific priority health issues is required during national public health strategy development. The themes need to reflect local factors in each nation, for example, national politics, historical national customs, and or an understanding of what is valued in a public health strategy. A thorough understanding by stakeholders of national issues of importance, combined with international learnings on the ‘optimal’ national public health strategy, ensures the development of a bespoke national public health strategy that is tailored to the citizens of that country.
- Governance of a national public health strategy should be robust and widely communicated to ensure effective strategy implementation. Within the current review, national governments, ministers or health ministries, and government-mandated bodies with public health responsibilities were identified as retaining overall accountability for national public health strategies. However, implementation of the strategy requires ownership, leverage and buy-in beyond central government at regional and local levels. Given political change over time, it is recommended that appropriate governance, with authority to control and with time for strategy implementation, is ensured.
- Evaluation of the implementation of a national public health strategy requires good access to multiple sources of both quantitative and qualitative data to attribute outcomes at population level. This requires the availability of robust health information systems within each nation. While seven countries included within the current review outlined KPIs which would be monitored to evaluate strategy impact, the number of KPIs available to each country varied significantly.
- The development of national public health strategies requires evidence–based collaborative approaches involving stakeholders across multiple sectors. Stakeholder engagement can be achieved using a variety of methods which includes both public consultation and targeted consultation with experts and representatives from governmental and non-governmental organizations at local, regional and national levels. Input from vulnerable groups at higher risk of health inequity or health inequality is particularly important.
