# Supplementary material for: A review of national public health strategies in selected countries
Source: Front Public Health. 2025 Mar 18;13:1391795. doi: 10.3389/fpubh.2025.1391795 (PMC11959016; doi:10.3389/fpubh.2025.1391795)
Supplement: Supplementary file 1 [file Data_Sheet_1.pdf]

Supplementary Material

A review of national public health strategies in selected countries.

***Michelle Norris[1], Eimear Burke[1,2], Fearghal Comaskey[1], Máire A. Connolly[3], Yvonne Doyle MD [4], Carol McLoughlin[1], Leah McManus[1], Valerie Power[1], Susan Spillane[1], †Máirín Ryan[1,5], †Michelle O'Neill[1].**

[1]Health Information and Quality Authority, Smithfield, Dublin 7, Ireland.

[2]Public Health Area E, Health Service Executive (HSE), Mount Kennett House, Henry Street Limerick, Ireland.

[3]School of Health Sciences, College of Medicine, Nursing and Health Sciences, University of Galway, Ireland.

[4]Visiting Professor at UCL Research Department of Epidemiology and Public Health, University College London, Gower Street, London, England, WC1E 6BT.

[5]Department of Pharmacology & Therapeutics, Trinity College Dublin, Trinity Health Sciences, James Street, Dublin 8, Ireland.

***Correspondence: Michelle Norris**
mnorris@hiqa.ie

†Last authorship is shared.

Table 1. List of countries and associated resources.

**Country**	**Resource(s)**
**EU/EEA**	
Austria	▪ Social Affairs, Health, Care and Consumer Protection<br>▪ Austrian Health Targets<br>▪ Austrian National Public Health Institute
Czechia	▪ Ministry of Health
Finland	▪ Ministry of Social Affairs and Health<br>▪ Finnish Institute for Health and Welfare
France	▪ The Ministry of Health and Prevention<br>▪ Public Health France
Portugal	▪ Directorate General of Health (DGS)
Spain	▪ Ministry of Health, Consumer Affairs and Social Welfare
Sweden	▪ Ministry of Health and Social Affairs<br>▪ Public Health Agency of Sweden
**Non-EU**	
Australia	▪ Department of Health and Aged Care
Canada	▪ Health Canada<br>▪ Public Health Agency of Canada
England	▪ Department of Health and Social Care<br>▪ Office for Health Improvement and Disparities<br>▪ UK Health Security Agency
New Zealand	▪ Ministry of Health - Manatū Hauora<br>▪ Health New Zealand – Te Whatu Ora
Northern Ireland	▪ Department of Health<br>▪ Health and Social Care Northern Ireland
Scotland	▪ Public Health Scotland
Singapore	▪ Ministry of Health
Wales	▪ Public Health Wales

Table 2. List of essential public health functions (EPHF), as defined by the WHO (1).

**Essential**
Monitoring and evaluating the population's health status, health service utilization and surveillance of risk factors and threats to health.
Public health emergency management.
Assuring effective public health governance, regulation and legislation.
Supporting efficient and effective health systems and multi sectoral planning, financing and management for population health.
Protecting populations against health threats, including environment and occupational hazards, communicable disease threats, food safety, chemical and radiation hazards.
Promoting prevention and early detection of diseases, including non communicable and communicable diseases.
Promoting health and well-being and actions to address the wider determinants of health and inequity.
Ensuring community engagement, participation and social mobilization for health and well-being.
Ensuring adequate quantity and quality of public health workforce.
Assuring quality of and access to health services.
Advancing public health research.
Ensuring equitable access to and rational use of essential medicines and other health technologies.

Table 3. Ten features which facilitate strategy translation to public health practice, as outlined in the Oxford Textbook of Global Public Health (2).

Number	Feature
**1**	Focus on populations rather than individuals.
**2**	Address the social determinants of health and their impact on poverty.
**3**	Be based on values of social justice and equity.
**4**	Reflect a global perspective.
**5**	Be aware of the dynamic relationship between the environment and health.
**6**	Place priority on prevention rather than curative care.
**7**	Understand the importance of systems and structures.
**8**	Take a transdisciplinary and multisectoral approach.
**9**	Ensure decision making is based on: (a) vital statistics (population-based data); (b) data, scientific evidence, and good research; and (c) surveillance and outbreak investigation.
**10**	Be committed to advocacy and in tune with politics.

Table 4. Sample data extraction template.

**Country (Reference)**	**Strategy information**
**Author(s)**<br>**Title**	
**Timeline**	
**Overall aim(s) (measurement method(s) and target(s) where available)**	
**Governance**	
**Scope and collaboration**	
**Themes and or priorities**	
**Implementation action(s), lead(s) and key performance indicator(s)**	
**Supporting economic analysis**	
**Any additional information**	
Strategy development	
**Stakeholder(s) and or consultation method(s)**	

Table 5. Aims and timeline identified in the national public health strategies of selected countries.

**Country**<br>**Information Source**<br>**Timeline**	**Aims**
**Australia**<br>National Preventive Health Strategy: Valuing health before illness: Living well for longer (3)<br>2021-2030	To improve the health and wellbeing of all Australians at all stages of life through prevention.<br><br>4 further aims (with associated targets) are also outlined:<br>1. All Australians have the best start in life.<br>This Strategy recognises the value of health and wellbeing at all stages of life, which emphasises the significance of prevention from preconception period through to the early years of life.<br>Target(s):<br>▪ the proportion of the first 25 years lived in full health will increase by at least 2% by 2030<br>▪ the proportion of Aboriginal and Torres Strait Islander babies with a healthy birthweight will increase to at least 91% by 2031<br>▪ the proportion of the first 0-4 years of life lived in full health will increase by at least 3.5% by 2030.<br><br>2. All Australians live in good health and wellbeing for as long as possible.<br>A strong focus on preventive health and health promotion can extend the quality of life and life expectancy of Australians.<br>Target(s):<br>▪ Australians will have at least an additional 2 years of live lived in full health by 2030.<br><br>3. Health equity is achieved for priority populations.<br>The burden of ill-health is not shared equally amongst Australians. This Strategy will result in overall greater gains for parts of the Australian community who are burdened unfairly due to the wider determinants of health.<br>Target(s):<br>▪ Australians in the 2 lowest Socio-Economic Index For Areas (SEIFA) quintiles will have at least an additional 3 years of life lived in full health by 2030<br>▪ Australians in regional and remote areas will have at least an additional 3 years of life lived in full health by 2030<br>▪ Aboriginal and Torres Strait Islander people will have at least an additional 3 years of life lived in full health by 2030.<br><br>4. Investment in prevention is increased.<br>Investment in prevention needs to be enhanced in order to achieve a better balance between treatment and prevention in Australia.<br>Target(s):

Country<br>Information Source<br>Timeline	Aims
	▪ Investment in preventive health will rise to be 5% of total health expenditure across Commonwealth, state and territory governments by 2030.
**Austria**<br>▪ Austrian Health Targets – long healthy life years for all (4)<br>2012-2032<br><br>▪ ROADMAP "Future Health Promotion": 10 packages of measures for a health-promoting future in Austria" (5)<br>2023-2027	▪ To prolong the healthy lives of all people living in Austria irrespective of their level of education, income or personal living conditions.<br><br>▪ To formulate and prioritise measures for better health and quality of life for all people living in Austria, in the next 5 years.
**Canada**<br>Creating a Healthier Canada: Making Prevention a Priority A Declaration on Prevention and Promotion from Canada's Ministers of Health and Health Promotion/Healthy Living (6)<br>2010 onwards	To improve health and reduce health disparities and to build and influence the physical, social and economic conditions that will promote health and wellness, and prevent illness so that Canadians can enjoy good health for years to come. This will be achieved through the government working together and with private, non-profit, municipal, academic and community sectors, and with First Nations, Inuit and Métis peoples.
**England**<br>Public Health Outcomes Framework (PHOF) (7)<br>2013 onwards	The PHOF sets out a high-level overview of public health outcomes, at national and local level, supported by a broad set of indicators. The vision is to improve and protect the nation's health and wellbeing, and improve the health of the poorest, fastest.
**Finland**<br>Promoting well-being, health and safety in 2030: Decision in principle of the Government (8)<br>2021-2030	To secure the sustainable well-being of Finns in all populations and age groups. The decision outlines the long-term measures that will reduce the inequality in the well-being and health of Finns, by 2030.
**Northern Ireland**<br>Making life better: A whole system strategic framework for public health (9)<br>2013-2023	To achieve better health and wellbeing for everyone and reduce inequalities in health. This corresponds with the vision that all people are enabled and supported in achieving their full health and wellbeing potential.
**Portugal**<br>National Health Plan (PNS) Sustainable Health: from everyone to everyone (10)<br>2021-2030	To improve the health and well-being of the population throughout the life cycle, through a social commitment to health without leaving anyone behind, while preserving the planet and without compromising the health of future generations.
**Scotland**	

**Country**<br>**Information Source**<br>**Timeline**	**Aims**
▪ National Performance Framework (NPF) (11)<br>2007 onwards<br><br>▪ Public Health Priorities for Scotland (12)<br>2018 onwards	▪ The framework aims to:<br>○ create a more successful country<br>○ give opportunities to all people living in Scotland<br>○ increase the wellbeing of people living in Scotland<br>○ create sustainable and inclusive growth<br>○ reduce inequalities and give equal importance to economic, environmental and social progress.<br><br>▪ To improve the health of the population and to reduce the unacceptable variation in life expectancy that exists across Scotland.
**Spain**<br>Public Health Strategy (ESP) 2022: Improving the Health and Well-Being of the Population (13)<br>2022-2026	To lay the foundation for strengthening Spain's public health system and provide it with a roadmap that aims to guarantee the full exercise of the population's right to health.
**Sweden**<br>▪ Good and equal health – a developed public health policy (14)<br>2018<br><br>▪ Towards good and equal health: A framework for implementing and monitoring the national Public Health policy (15)<br>2021	▪ To eliminate avoidable health inequalities within one generation.<br><br>▪ To enable systematic and coordinated efforts that support the realisation of this national public health goal.

Table 6. Scope and strategy alignment of the national public health strategies of selected countries.

Country<br>Information Source	Scope and strategy alignment
**Australia**<br>National Preventive Health Strategy: Valuing health before illness: Living well for longer (3)	The responsibility for creating positive change by 2030 is shared by: all governments, the non-government sector, research and academia, the private sector, industries, communities and individuals. Achieving strategy goals will require close alignment with other key areas of health reform for the Australian Government (the Primary Health Care 10 Year Plan and the National Aboriginal and Torres Strait Islander Health Plan). It will also require alignment with other whole-of-government approaches, including the new National Agreement on Closing the Gap.<br><br>**United Nations (UN) Sustainable Development Goals (SDGs) and or WHO Targets**<br>The strategy outlined 3 of 4 aims which aligned with UN SDGs and WHO targets.<br>1. All Australians have the best start in life<br>This aligns broadly with:<br>▪ UN SGD Target 3.1<br>▪ UN SGD Target 3.2<br>▪ UN SDG Target 3.7<br>▪ UN SDG Target 5.6<br>▪ WHO 'Healthier Populations' triple billion goal<br>▪ WHO 'Universal Health Coverage' triple billion goal<br>▪ WHO Global nutrition Targets 2025<br><br>2. All Australians live in good health and wellbeing for as long as possible<br>This aligns broadly with:<br>▪ UN SDG Target 3.8<br>▪ WHO 'Healthier Populations' triple billion goal<br><br>3. Health equity is achieved for priority populations.<br>This aligns broadly with:<br>▪ UN SDG Target 3.8<br>▪ WHO 'Healthier Populations' triple billion goal<br><br>The Framework for Action forms the foundation for this strategy and contains 3 interlinked elements. Targets within one of these elements, '**Boosting action focus areas- accelerating action in initial priority areas and evolving to address future needs'** have been identified to align directly or broadly with UN SDGs or WHO targets.
**Austria**<br>▪ Austrian Health Targets – long healthy life years for all (4)	

**Country**<br>**Information Source**	**Scope and strategy alignment**
▪ ROADMAP “Future Health Promotion": 10 packages of measures for a health-promoting future in Austria” (5)	▪ The health goals focus on multisectoral factors that have a decisive influence on health, such as education, work situation, social security or environmental influences (see section 3.2.3 Themes or priorities, **Error! Reference source not found.**).<br><br>**UN SDGs and or WHO Targets**<br>Of the 10 Health Goals outlined in the strategy, one of these goals aligned with a WHO target. This was Health goal 4: Secure air, water, soil and all habitats for future generations which was implemented as a result of the “Environment and Health in Europe" process of the WHO Euro and the Economic and Social Committee for Europe.<br><br>▪ The proposed package is intersectoral as is displayed in themes and priorities which lie across health, social and environmental domains (see section 3.2.3 Themes or priorities, **Error! Reference source not found.**).
**Canada**<br>Creating a Healthier Canada: Making Prevention a Priority A Declaration on Prevention and Promotion from Canada’s Ministers of Health and Health Promotion/Healthy Living (6)	None identified.
**England**<br>Public Health Outcomes Framework (PHOF) (7)	The PHOF indicators were located across 4 domains; improving the wider determinants of health; health improvement; health protection; and healthcare and premature mortality.
**Finland**<br>Promoting well-being, health and safety in 2030: Decision in principle of the Government (8)	The decision in principle implements the ministries' strategic goals and strengthens welfare economic thinking. It is part of the implementation of the UN Sustainable Development Goals (Agenda 2030) in Finland.<br><br>Collaboration between ministries and their subordinate agencies and institutions, other research and development institutions, advisory boards, municipalities, regional operators, organisations, educational organisations, companies and other service providers occurs during implementation. Implementation is carried out as part of the activities of various administrative branches and organisations. The ministries are responsible in their own administrative area for the implementation of the measures (see section 3.2.4 Implementation, Table 3.6).
**Northern Ireland**<br>Making life better: A whole system strategic framework for public health (9)	The strategy focuses on the broad range of social, economic and environmental factors which influence health and wellbeing. It brings together actions at government level and provides direction for implementation at regional and local level.<br><br>At strategic level this framework emphasises the inter-connectedness of many government policies and programmes, and the mutual benefits and shared goals that can be achieved by working together effectively. It is clear that there are opportunities to

Country<br>Information Source	Scope and strategy alignment
	strengthen these linkages through governance and monitoring which develops a sense of coherence flowing through to implementation at delivery level.<br><br>The reform of local government will also provide an opportunity to strengthen the already significant contribution at local level to improving health and reducing health inequalities. The productive joint working arrangements between the Public Health Agency and councils will be maintained and built upon, as well as ensuring strong linkages with others through the new community planning process.<br><br>A whole system approach is required, with clear lines of communication, accountability and clarity on how governance and implementation is to work. Connections with other relevant strategies and initiatives need to be managed and maximised. Collaboration should be embedded in every aspect of governance and monitoring, and with clear recognition of and relevant linkage with structures and partnerships which will contribute - examples are Children and Young People's Strategic Partnership, and Public Health Local Government Steering Group.
**Portugal**<br>National Health Plan (PNS) Sustainable Health: from everyone to everyone (10)	The plan favours a "whole of government" approach (and not only from the ministry of health) and from "all of society" (and not just from the health sector). Multisector collaboration is essential to achieve better sustainable health.<br><br>Major strategic guidelines for the PNS 2021-2030 plan include:<br>▪ Investing in the health promotion and prevention: This includes the health promotion in the many areas in society, such as schools and workplaces, and promoting healthy behaviours such as breastfeeding, healthy eating and physical activity. Prevention of health risks such as environmental risks and alcohol, tobacco and illicit psychoactive substances.<br>▪ Universal Health Coverage: This includes SNS and health for all, leaving no one behind and highlighting Health Promotion, Health Protection, Disease Prevention and Caregiving.<br>▪ Innovation: This includes the support of research aligned with sustainable health and the development of precision public health.<br>▪ Prepare and anticipate the future: This includes continuous development/adjustment of plans, on a prospective basis, in order to quickly adapt intervention strategies to "new" health needs. And to develop "instrumental areas" such as human resources, infrastructure and information and communication technologies. Adaptation of health promotion and protection strategies according to the needs and expectations of the population. Investment in patient safety (including the prevention of "new" resistance to antimicrobials, the avoidance of polypharmacy, especially in the elderly, and the excessive use of medication in mental health among others).
**Scotland**<br>▪ National Performance Framework (NPF) (11)	▪ To achieve the national outcomes, the NPF aims to get everyone in Scotland to work together. This includes:<br>– national and local government<br>– businesses<br>– voluntary organisations<br>– people living in Scotland

Country<br>Information Source	Scope and strategy alignment
▪ Public Health Priorities for Scotland (12)	**UN SDGs and WHO Targets**<br>The NPF and the SDGs share the same aims. The NPF is Scotland's way to localise the SDGs. The NPF has a focus on tackling inequalities so that no one in Scotland is left behind as goals are achieved. The SDG Network Scotland is an open coalition bringing together the voices of over 500 people and organisations across Scotland to assist with the development of a Scotland-wide response to the challenge set by the SDGs. The Scottish Government and the Convention of Scottish Local Authorities (COSLA) work closely with the SDG Network Scotland.<br><br>▪ The priorities connect strongly to, and accelerate, the Scottish Government and COSLA's wider work and include local strategic planning and partnership activity; the refreshed NPF and related National Outcomes; the Digital Health and Care Strategy, and further public health policies, and efforts towards sustainable economic growth.<br>The priorities are a foundation for the whole system, for public services, third sector, community organisations and others, to work better together to improve Scotland's health, and to empower people and communities. They are a starting point for new preventative approaches, and a new awareness around wellbeing, that will develop and strengthen in the coming years.
**Spain**<br>Public Health Strategy (ESP) 2022: Improving the Health and Well-Being of the Population (13)	The strategy is a cross-cutting and integrative approach and is based on work on health determinants, health in all policies, the "One Health" approach and governance for health. It is also aligned with the 2030 UN SDGs, as a commitment to the necessary improvement of global health.<br><br>It requires the involvement of society as a whole, the necessary participation and collaboration of:<br>▪ all levels of Public Administration (national, regional and local)<br>▪ scientific and social entities<br>▪ patients' associations and non-governmental groups<br>▪ community participation through individual or group actions.<br><br>**UN SDGs and WHO Targets**<br>Strategic lines and actions have been identified and also align with the UN SDGs.
**Sweden**<br>Towards good and equal health: A framework for implementing and monitoring the national Public Health policy (15)	The framework was developed for the implementation and monitoring of the national public health policy which emphasised:<br>▪ health inequity is a societal problem and hampers sustainable development<br>▪ public health is a shared responsibility for all sectors at all levels, requiring actions by public and private sectors, as well as civil society and individuals.<br>▪ all sectors should endeavour to promote good and equitable health<br>▪ health in all policies should guide work in all sectors at the local, regional, and national levels<br>▪ collaboration at the national level can facilitate public health work at regional and local levels.

Table 7. Themes or priorities identified in the national public health strategies of selected countries.

Country<br>Information Source	Themes or priorities
**Australia**<br>National Preventive Health Strategy: Valuing health before illness: Living well for longer (3)	7 principles were outlined to underpin the Framework for Action:<br>▪ The equity lens<br>▪ Multi-sector collaboration<br>▪ Embracing the digital revolution<br>▪ Enabling workforce<br>▪ Community participation<br>▪ Empowering and supporting Australians<br>▪ Adapting to emerging threats and evidence.
**Austria**<br>▪ Austrian Health Targets – long healthy life years for all (4)	▪ 10 Health Goals were developed in 2012 and form the framework for action for an overall health-promoting policy:<br>Health Goal 1: Working together to create health-promotional living and working conditions.<br>Health Goal 2: Ensure equal health opportunities for everyone in Austria.<br>Health Goal 3: Strengthen the health literacy of the population.<br>Health Goal 4: Secure air, water, soil and all habitats for future generations.<br>Health Goal 5: Strengthen health through social cohesion.<br>Health Goal 6: Ensure that children and young people grow up healthy in the best possible way.<br>Health Goal 7: Make healthy and sustainable nutrition accessible to all.<br>Health Goal 8: Promote healthy and safe exercise in everyday life.<br>Health Goal 9: Promote psychosocial health.<br>Health Goal 10: Ensure high quality and affordable healthcare for all.
▪ ROADMAP "Future Health Promotion": 10 packages of measures for a health-promoting future in Austria (5)	▪ 10 bundles of measures for a future with a good quality of life, with many healthy years of life and equal opportunities for all people living in Austria, are recommended:<br>– Social society and ecological management: Promote socially and ecologically compatible business practices, taking equal opportunities into account<br>– Health and social affairs: Reorientation in health and social services as well as in nursing towards more health promotion and comprehensive prevention<br>– Climate resilient and movement-enhancing living and social spaces: Promote movement and respectful, solidary coexistence in climate-resilient and environmentally friendly living spaces<br>– Workplace: Promote integrated company health management and support small (or very small) companies in particular in ensuring a health-promoting working environment<br>– Psychosocial Wellbeing: Promote psychosocial health, the elimination of taboos on mental stress and respectful cooperation<br>– Grow up: Design educational and care rooms for children, young people and educators in a health-promoting manner

Country<br>Information Source	Themes or priorities
	– Sustainable food system: Creating a healthy, fair and sustainable food system, especially in communal catering<br>– Resources for health promotion: Increase financial and human resources, strengthen structures and knowledge about health promotion, improve cooperation<br>– Participation of population: Strengthen the participation of the population as the key to better health<br>– Health literacy and information: Prepare health information in a target group specific and easily accessible manner and make it (digitally) accessible.
**Canada**<br>Creating a Healthier Canada: Making Prevention a Priority. A Declaration on Prevention and Promotion from Canada's Ministers of Health and Health Promotion/Healthy Living (6)	5 principles were outlined in the Declaration:<br>– Prevention is a priority.<br>– Prevention is a hallmark of a quality health system.<br>– Prevention is the first step in management.<br>– Health promotion has many approaches that should be used.<br>– Health promotion is everyone's business.
**England**<br>Public Health Outcomes Framework (PHOF) (7)	None identified.
**Finland**<br>Promoting well-being, health and safety in 2030: Decision in principle of the Government (8)	4 focal points were outlined:<br>1. Opportunity for everyone to participate.<br>2. Good everyday environments.<br>3. Activities and services that promote well-being and health.<br>4. Effectiveness with decision making.
**Northern Ireland**<br>Making life better: A whole system strategic framework for public health (9)	2 underpinning themes were identified:<br>▪ sustainable communities<br>▪ building health public policy.<br><br>6 main themes were then identified:<br>1. Giving every child the best start.<br>2. Equipped throughout life.<br>3. Empowering healthy living.<br>4. Creating the conditions.<br>5. Empowering communities.<br>6. Developing collaboration.
**Portugal**<br>National Health Plan (PNS) 2021-2030. Sustainable Health: from everyone to everyone (PNS 2021-2030) (10)	5 major goals and 15 strategic objectives were identified:<br>1. Reduce inequalities:<br>▪ promote health equity<br>▪ promoting peace, justice and prosperity<br>▪ promote partnerships between all sectors of society

Country<br>Information Source	Themes or priorities
	2. Promote the development of healthy behaviours, cultures and communities:<br>▪ promote health literacy<br>▪ streamline health-promoting environments<br>▪ promote longevity and active and healthy ageing<br><br>3. Minimise the consequences of climate change and other environmental determinants on health:<br>▪ protect the planet for present and future generations<br>▪ streamline environmental risk surveillance systems and associated problems<br>▪ ensure preparedness and response in public health emergencies<br><br>4. Reduce the burden of communicable and non-communicable diseases in an integrated way:<br>▪ Strengthen sustainable healthcare<br>▪ Strengthen access to quality healthcare<br>▪ Streamline the integration of person-centred care<br><br>5. Keeping health problems currently under control:<br>▪ Ensure sexual/reproductive, maternal and child health surveillance and quality of care<br>▪ Maintain a high level of immunisation coverage<br>▪ Keeping water-borne health problems under control
**Scotland**<br>▪ National Performance Framework (NPF) (11)	▪ The national outcomes are:<br>o Children and young people: we grow up loved, safe and respected so that we realise our full potential<br>o Communities: we live in communities that are inclusive, empowered, resilient and safe<br>o Culture: we are creative and our vibrant and diverse cultures are expressed and enjoyed widely<br>o Economy: we have a globally competitive, entrepreneurial, inclusive and sustainable economy<br>o Education: we are well educated, skilled and able to contribute to society<br>o Environment: we value, enjoy, protect and enhance our environment<br>o Fair work and business: we have thriving and innovative businesses, with quality jobs and fair work for everyone<br>o Health: we are healthy and active<br>o Human rights: we respect, protect and fulfil human rights and live free from discrimination<br>o International: we are open, connected and make a positive contribution internationally<br>o Poverty: we tackle poverty by sharing opportunities, wealth and power more equally.
▪ Public Health Priorities for Scotland (12)	▪ 6 priorities were outlined:<br>Priority 1: A Scotland where we live in vibrant, healthy and safe places and communities<br>Priority 2: A Scotland where we flourish in our early years

Country<br>Information Source	Themes or priorities
	Priority 3: A Scotland where we have good mental wellbeing<br>Priority 4: A Scotland where we reduce the use of and harm from alcohol, tobacco and other drugs<br>Priority 5: A Scotland where we have a sustainable, inclusive economy with equality of outcomes for all<br>Priority 6: A Scotland where we eat well, have a healthy weight and are physically active.
**Spain**<br>Public Health Strategy (ESP) 2022: Improving the Health and Well-Being of the Population (13)	4 strategic lines of action were outlined:<br><br>Strategic Line 1: Strengthening public health to improve the health of the population.<br>Strategic Line 2: Update public health surveillance and ensure response capacity to health risks and emergencies.<br>Strategic Line 3: Improving health and well-being through the promotion of health, safe and sustainable lifestyles and environments.<br>Strategic Line 4: Promote health and health equity throughout the life course.
**Sweden**<br>Towards good and equal health: A framework for implementing and monitoring the national Public Health policy (15)	The national Public Health policy identified 8 target areas.<br><br>**Target and Focus Areas**<br>1. <u>Conditions in early life:</u><br>▪ equal maternal and child healthcare<br>▪ an equivalent preschool of high quality<br>▪ method and means that put the children first in focus.<br><br>2. <u>Knowledge, skills and education/training:</u><br>▪ a good learning environment at school<br>▪ an equal education system<br>▪ countering school failure through early identification and intervention.<br><br>3. <u>Work, working conditions and work environment:</u><br>▪ to have a job<br>▪ good conditions for increased employability<br>▪ good work and employment conditions<br>▪ a physically and psychosocially sustainable work environment.<br><br>4. <u>Income and economic resources:</u><br>▪ distribution of income in the population<br>▪ financial resources for those with tight margins and reduced long-term financial vulnerability.

Country<br>Information Source	Themes or priorities
	5. Housing and neighbourhood conditions:<br>▪ access to a fully functional and affordable home<br>▪ residential areas that are socially sustainable<br>▪ healthy living environments on equal terms.<br><br>6. Health behaviours:<br>▪ increased accessibility to health-promoting products, environments and activities<br>▪ to strengthen the health promotion and prevention work with lifestyles in welfare organisations.<br><br>7. Control, influence and participation:<br>▪ equal participation in democracy<br>▪ equal participation in civil society<br>▪ the work for human rights<br>▪ sexual and reproductive health and rights<br>▪ increased control, influence and participation for certain individuals and groups.<br><br>8. Equitable and health-promoting health and medical services:<br>▪ accessibility to meet different needs<br>▪ care meetings that promote health and create conditions for equal efforts and results<br>▪ the health promotion and preventive work of the health and medical services<br>▪ good, accessible and equal dental care<br>▪ that regions lead, control and organise their activities with the aim of providing conditions for equal care.

Table 8. Governance identified in the national public health strategies of selected countries.

**Country**<br>**Information Source**	**Governance**
**Australia**<br>National Preventive Health Strategy: Valuing health before illness: Living well for longer (3)	Enhanced governance structures are required to create a more resilient prevention system. This includes:<br>▪ an independent, expert-led mechanism that will advise the Australian Government on current, emerging and future priorities in prevention<br>▪ a governance mechanism within government, and across relevant portfolios, that have an influence on the health and wellbeing of Australians.
**Austria**<br>▪ Austrian Health Targets – long healthy life years for all (4)<br><br>▪ ROADMAP "Future Health Promotion": 10 packages of measures for a health-promoting future in Austria (5)	▪ **Federal Ministry for Social Affairs, Health, Care and Consumer Protection:** head of process coordination, technical and organisational support.<br>**Health Austria:** technical and organisational support.<br><br>▪ None identified.
**Canada**<br>Creating a Healthier Canada: Making Prevention a Priority A Declaration on Prevention and Promotion from Canada's Ministers of Health and Health Promotion/Healthy Living (6)	None identified.
**England**<br>Public Health Outcomes Framework (PHOF) (7)	None identified.
**Finland**<br>Promoting well-being, health and safety in 2030: Decision in principle of the Government (8)	The monitoring of the implementation plan is coordinated by the Public Health Advisory Board, which consists of experts from ministries and various administrative sectors. The ministries are responsible in their own administrative area for ensuring the decision in principle will be implemented.
**Northern Ireland**<br>Making life better: A whole system strategic framework for public health (9)	**Ministerial Committee for Public Health**: chaired by the Minister for Health, Social Services and Public Safety and supported and informed by the All Departments Officials Group (ADOG). Key functions are:<br>▪ to provide strategic leadership, direction and coherence with other key strategic programmes and structures such as Programme for Government, NI Economic Strategy and Delivering Social Change,<br>▪ agree shared goals and priorities and oversee implementation on behalf of the Executive.<br><br>**The ADOG**: chaired by the Chief Medical Officer, will comprise senior officials from all departments. Key functions are to:<br>▪ inform and make recommendations to the Ministerial Committee<br>▪ co-ordinate collaborative working at departmental level

Country<br>Information Source	Governance
	▪ connect with the Regional Project Board, directing, or supporting action as appropriate<br>▪ monitor and report on progress.<br><br>**The Regional Project Board**: led by the Public Health Agency will focus on strengthening collaboration and co-ordination to deliver on shared strategic priorities across sectors at a regional level, and on supporting implementation at a local level. Membership of the group will comprise the Chief Officers of relevant statutory agencies, and include representation from local government, the community and voluntary sector and the private sector. The primary focus of this group will be to drive implementation of agreed priorities through:<br>▪ building connections between strategic drivers and local implementation<br>▪ driving forward opportunities for regional initiatives that cut across common themes<br>▪ directing, providing co-ordination for and monitoring the work of local partnerships<br>▪ examination of emerging data, evidence and best practice in terms of addressing health and social wellbeing inequalities<br>▪ providing advice and recommendations to the ADOG and Ministerial Committee on emerging issues and potential areas for policy and legislative consideration and joint working.<br><br>The Regional Project Board may be supported through sub-groups or time bound working groups on priority themes. The Group will report through the Chair to the ADOG. Individual members will also be required to make effective links into their relevant Department or organisation in terms of emerging issues and implementation. In conjunction with local level partnerships the Regional Project Board will develop an Implementation Plan.<br><br>**Local strategic partnerships:** of key statutory, private, community and voluntary bodies will be established based on an agreed geographic coverage. Each Partnership should in the first instance be developed from existing local arrangements and include a balance of statutory and non-statutory partners.<br>The partnerships' role will focus on local delivery and will be to:<br>▪ identify local opportunities for collaboration and partnership working based on local need<br>▪ drive local interventions/services to support those most in need<br>▪ develop and promote new ways of working and models of intervention and test concepts<br>▪ ensure regional priorities are reflected in local plans<br>▪ ensure that local priorities are fed into the strategic process<br>▪ report to the Regional Project Board (the Chair of the local partnership will be a member of the Regional Project Board).

Country<br>Information Source	Governance
	**Community Planning**: legislation places a duty on councils to lead the community planning process and on other public bodies to participate. Departments will be required to promote and encourage community planning and have regard to the councils' community plans in planning the delivery of services.<br>**Data and Research Groups:** established to support the framework, a set of key indicators has been agreed to facilitate high-level monitoring of progress.<br>**DHSSPS Information and Analysis Directorate:** will undertake the role of collating and publishing updates on the key indicators including on those relating to the social determinants.<br><br>Monitoring<br>To support the proposed structures a monitoring framework will be developed to include:<br>▪ reports from local partnerships to the Regional Project Board<br>▪ reports from the Regional Project Board on regional and local activity with advice and recommendations to ADOG<br>▪ reports from ADOG to the Ministerial Committee on strategic issues, key indicator trends, overall activity and to provide advice and recommendations<br>▪ an annual report on overall progress.
**Portugal**<br>National Health Plan (PNS) 2021-2030. Sustainable Health: from everyone to everyone (10)	**The Basic Health Law:** defines as one of the main foundations of the health policy the National Health Plan.<br>**Ministry of Health:** responsible for the technical coordination of implementation and evaluation. The Ministry of Health will also manage the alignment of strategic options for health organisations and services with the population-based health plans, namely with regard to identified health needs, recommend support mechanisms and select health strategies at a national and subnational level.<br>**The Directorate-General for Health (DGS):** responsible for coordinating in the areas of planning strategy in health and ensuring the elaboration and execution of PNS 2021-2030.<br>**National Health Institute Dr. Ricardo Jorge:** responsible for the evaluation of the implementation of the PNS 2021-2030 and the results.
**Scotland**<br>▪ National Performance Framework (NPF) (11)<br><br>▪ Public Health Priorities for Scotland (12)	▪ **Scottish Government**: will focus activities and spending to help meet National Outcomes. They will work with the wider public sector and others to help them work in a way that meets these outcomes.<br>**Local government**: work with the Scottish Government and other organisations in their community.<br>**Community Planning Partnerships:** bring together organisations in Scotland. They will then look to work on local improvement plans.<br><br>▪ None identified.
**Spain**<br>Public Health Strategy (ESP) 2022: Improving the Health and Well-Being of the Population (13)	**The State Centre for Public Health:** the body responsible for technical and scientific advice on public health, the evaluation of interventions, strategy monitoring and evaluation, as well as the coordination of the actions developed by the national public health centres.

Country<br>Information Source	Governance
	**Ministry of Health:** the approach and the strategic lines of ESP 2022 will be developed under the auspices of the Ministry of Health and within the framework of the Inter-territorial Board of the National Health System, a body for co-governance and comparison of proposals from all public agents with competence in the health sector.
**Sweden**<br>Towards good and equal health: A framework for implementing and monitoring the national Public Health policy (15)	The strategy is built on current governance structures and the division of responsibilities between government agencies, as well as the legally-required cooperation between them. There are no clear contact points for overall public health issues among the government authorities. However, joint inter-agency dialogue on priorities for public health work is deemed adequate and provides the opportunity for shared responsibility instead of additional regulation. The Public Health Agency have outlined there is no need to formalise the coordination.

Table 9. Implementation actions, agencies and key performance indicators identified in the national public health strategies of selected countries.

Country<br>Information Source	Implementation action(s), agency(ies) and key performance indicator(s)
**Australia**<br>National Preventive Health Strategy: Valuing health before illness: Living well for longer (3)	The Framework for Action forms the foundation of the Strategy, providing a strategic and structured approach to achieve better health and wellbeing for all Australians by 2030. The Framework is composed of 3 interlinked elements:<br>1. Mobilising a prevention system<br>2. Boosting action in focus areas<br>3. Continuing strong foundations (not outlined below as is continuation of ongoing strategies and work).<br><br>**Mobilising a prevention system**<br>Seven system enablers are identified in the strategy that are critical to creating a more effective and integrated prevention system for Australia over the next 10 years. One desired policy achievement has been outlined below for each system enabled.<br><br>**1. Leadership, governance & funding**<br>The priorities for preventive health action are informed by a national, independent governance mechanism that is based on evidence, effectiveness and relevance.<br>**2. Prevention in the health system**<br>Increased investment in resilient system infrastructure, particularly service models, workforce roles and capacities, digital health technologies and funding levers, enables preventive health to be embedded across the health system.<br>**3. Partnerships & community engagement**<br>Innovative partnerships are established between and within sectors that influence health, to ensure shared decision-making and to drive evidence-based change.<br>**4. Information & health literacy**<br>Consumers are informed by a national platform that provides or identifies credible, evidence-based health information.<br>**5. Research & evaluation**<br>A systematic approach to the prioritisation of preventive health research is established to address key gaps including the impact of the wider determinants of health.<br>**6. Monitoring & surveillance**<br>A preventive health governance mechanism supports the monitoring and surveillance of this Strategy.<br>**7. Preparedness**<br>A national strategic plan addressing the impacts of environmental health, including horizon scanning to identify and understand future threats, is developed and implemented in alignment with this Strategy and the work of the Environmental Health Standing.<br><br>**A number of enablers and desired achievements were then selected as <u>immediate priorities:</u>**<br>1. Governance mechanisms<br>2. Increased investment in prevention

Country<br>Information Source	Implementation action(s), agency(ies) and key performance indicator(s)
	3. A national platform providing credible and reliable health information<br>4. Embedding prevention in primary healthcare and aligning with the Primary Health Care 10 Year Plan<br>5. National consumer engagement strategy<br>6. National health literacy strategy<br>7. Enhanced public health workforce planning<br>8. Ongoing national data sets to support the monitoring and evaluation of this Strategy and a national prevention monitoring and reporting framework.<br><br>**Boosting action in focus areas – accelerating action in initial priority areas and evolving to address future needs**<br>▪ Reducing tobacco use and nicotine addiction<br>▪ Improving access to and the consumption of a healthy diet<br>▪ Increasing physical activity<br>▪ Increasing cancer screening and prevention<br>▪ Reducing alcohol and other drug harm<br>▪ Promoting and protecting mental health.
**Austria**<br>▪ Austrian Health Targets – long healthy life years for all (4)	▪ Measures along with desired outcomes and implementing institutions are outlined for each Health Goal. One measure is presented for each Health Goal below.<br><br>**Health Goal 1: Creating health-promoting living and working conditions together. Creating population groups through the cooperation of all areas of politics and society.**<br><u>Healthy and active aging dialogue:</u><br>**Desired outcome:** The dialogue connects organisations and actors in order to make activities visible, create synergies and spread knowledge. In dialogue, models of good practice are identified and widespread implementation is initiated. In dialogue, tools are developed that change the images of aging in our society for the better.<br>**Implementing institutions:** The dialogue is a cooperation between the Federal Ministry for Social Affairs, Health, Care and Consumer Protection, the umbrella organisation of Austrian social insurance and the Healthy Austria Fund.<br><br>**Health Goal 2: Ensure equal health opportunities for everyone in Austria.**<br><u>Increased establishment of early intervention:</u> "Frühe Hilfen" follows a multi-professional as well as multi-sectoral approach (investing in networking esp. with services from the health and social sector) to support families in raising healthy children. The programme is voluntary and ensures access of families through direct contact and through professionals, such as health and social workers, who can identify and refer them.<br>**Desired outcome:** Establish regional early intervention networks to relieve the burden on families as early as pregnancy and thus ensure a health start in life and more equal opportunities.

Country<br>Information Source	Implementation action(s), agency(ies) and key performance indicator(s)
	**Implementing institutions:** Federal Ministry of Social Affairs, Health, Care and Consumer Protection (Areas of Social Affairs and Health), Federal Chancellery (Areas of Family and Youth, Women and Gender Equality), Provinces and Social Insurance Institutions, Health Austria, Federal Ministry for Europe, Integration and Foreign Affairs, Health Austria Fund.<br><br>**Health Goal 3: Strengthen the health literacy of the population.**<br>Establishment of the Austrian Platform for Health Literacy<br>**Desired outcome:** The national development of health literacy.<br>**Implementing institutions:** Federal Ministry of Social Affairs, Health, Care and Consumer Protection, Social Insurance Institutions, Federal States, Federal Chancellery (Department of Families and Youth), Federal Ministry of Education, Science and Research, Federal Ministry of Arts, Culture, Civil Service and Sport, Healthy Austria Fund and multipliers.<br><br>**Health Goal 4: Secure air, water, soil and all habitats for future generations.**<br>**Desired outcomes:** The impact objectives are to:<br>– Maintain and strengthen the foundations for a healthy life through the responsible and sustainable use of resources and through the same design of the living space<br>– Avoid, identify, monitor and, if possible, reduce environmental pressures with potential health impacts<br>– Promote/strengthen awareness of the relationship between environment and health among the population and decision-makers and ensure environmental justice in the best possible way.<br>**Implementing institutions:** N/A.<br><br>**Health Goal 5: Strengthen health through social cohesion.**<br>**Desired outcomes:** The impact objectives are to:<br>– Extend opportunities for social participation and participation for all, thereby promoting inclusion<br>– Promote respect and solidarity between and for people and social groups in order to strengthen social cohesion in society<br>– Recognise diversity as an enrichment and challenge for society, taking into account the needs of minorities and disadvantaged population groups, and safeguarding and enforcing their rights.<br>**Implementing institutions:** N/A.<br><br>**Health Goal 6: Ensure that children and young people grow up healthy in the best possible way.**<br>Promotion of vocational training and labour market integration of young people<br>**Desired outcomes:** The extended compulsory training is intended to increase the chances of young people being able to participate sustainably and comprehensively in economic and social life.<br>**Implementing institutions:** Federal Ministry of Social Affairs, Health, Care and Consumer Protection / Ministry of Social Affairs, Federal Ministry of Labour, Federal Ministry for Digital and Economic Affairs.<br><br>**Health Goal 7: Make healthy and sustainable nutrition accessible to all.**

Country Information Source	Implementation action(s), agency(ies) and key performance indicator(s)
	**Desired outcomes:** The impact objectives of the working group are to:<br>– Create a cross-sectoral political, legal and economic framework for a sustainable food system that enables everyone to eat healthily<br>– Ensure a diverse, health-promoting and sustainable range of food and dishes<br>– Design the nutritional environment in a health-promoting and sustainable way.<br>**Implementing institutions:** N/A.<br><br>**Health Goal 8: Promote healthy and safe exercise in everyday life.**<br>Exercise at school: Many initiatives committed to promoting physical activity in schools. Examples include "Bewegte Klasse macht Schule" (Moving class makes school – automated translation) where interested schools are professionally accompanied and supported in the planning, implementation and sustainable anchoring of the topic of "movement" in the organised structure. Legal changes ensure that all-day school types include sufficient exercise in their leisure activities. This states that pupils should be given at least 5 exercise units per week.<br>**Desired outcomes:** Physical activity in schools.<br>**Implementing institutions:** Federal Ministry of Education, Science and Research and Lower Austrian Health and Social Fund – Initiative "Tut gut".<br><br>**Health Goal 9: Promote psychosocial health.**<br>Mental health in the work of smartphones<br>**Desired outcomes:** Poorer or less educated families may be at higher risk for these effects. The project aims to shed further light on these questions, focusing on the situation in Austria and limiting itself to the use of smartphones, as these are now widespread and available and usable anytime and anywhere, as well as a variety of possible uses (telephone, Internet, sending and receiving messages, communication via social media, photography, filming, listen to music, play games). Another focus is placed on the possible effects on mental health.<br>**Implementing institutions:** Institute for Health Promotion and Prevention, Vienna.<br><br>**Health Goal 10: Ensure high quality and affordable healthcare for all.**<br>Broad overarching goal with no specific measures.
▪ Health promotion strategy within the framework of the Federal Target Management Agreement (16)	▪ The health promotion strategy also serves as a support for the implementation of the Health Goals. It identifies 6 prioritised areas and allocates 66% of available funds to these areas. All prioritised areas are accompanied by impact targets and metrics:<br>1. Early help<br>2. Healthy crèches and health kindergartens<br>3. Healthy schools<br>4. Healthy living environments and healthy lifestyles for young people and people of working age<br>5. Health literacy of young people, people of working age and older people<br>6. Social participation and psychosocial health of older people.

Country<br>Information Source	Implementation action(s), agency(ies) and key performance indicator(s)
▪ ROADMAP "Future Health Promotion": 10 packages of measures for a health-promoting future in Austria (5)	▪ The recommended measures were assigned to 3 areas of intervention for the development of capacities in health promotion: Policy and networking, resources and implementation, and knowledge and skills. 1 action for each area of intervention is outlined below.<br><br>**Recommended actions:**<br>**Social society and ecological management**<br>▪ Policy and networking:<br>– Communicate how alternative indicators for measuring the success of a company may be used, such as how a company contributes to the preservation of ecological resources (for example, reduction of emissions) and to the promotion of social participation of vulnerable (for example, fair wages for gainful employment and the inclusion of vulnerable groups in the labour market).<br>▪ Resources and implementation:<br>– Expand supporting structures for care work (care and childcare) and promote other contact points which have low-threshold access and pre-requisite entry points within the municipals.<br>▪ Knowledge and skills:<br>– Further develop indicators, measurement and control instruments for a "Well-Being Society".<br><br>**Health and social care**<br>▪ Policy and networking:<br>– Adapt quality indicators in terms of health promotion.<br>▪ Resources and implementation:<br>– Guarantee structures for the participation of citizens and patients via contact points.<br>▪ Knowledge and skills:<br>– Collect data on healthcare workforce need.<br><br>**Climate resilient and movement-enhancing living and social spaces**<br>▪ Policy and networking:<br>– Intersectoral, legal anchoring of measures to adapt to climate change (including heat protection) and to protect the climate.<br>▪ Resources and implementation:<br>– Invest in health-promoting, exercise-friendly, traffic-calming urban and spatial planning (for example, by reducing parking space).<br>▪ Knowledge and skills:<br>– Communicate knowledge about the positive effects of regular exercise (for example, exercise recommendations) and encourage active mobility, exercise and sport.<br><br>**Workplace**<br>▪ Policy and networking:<br>– Clarify the tasks of all relevant stakeholders in Workplace Health Management (WHM) and actors and establish interface management for WHM.

Country<br>Information Source	Implementation action(s), agency(ies) and key performance indicator(s)
	▪ Resources and implementation:<br>– Provide coordinated inputs on WHM topics as part of vocational training.<br>▪ Knowledge and skills:<br>– Enabling and promoting a common understanding through campaigns and information on WHM.<br><br>**Psychosocial Wellbeing**<br>▪ Policy and networking:<br>– Develop a national mental health action plan.<br>▪ Resources and implementation:<br>– Ensure easy and quick access to preventive care, advice and therapy for mental stress and illnesses, especially for people with cognitive impairments.<br>▪ Knowledge and skills:<br>– Strengthen health-promoting communication and personal development, among other things, at school (for all school partners).<br><br>**Grow up**<br>▪ Policy and networking:<br>– Revise or "clear out" educational framework plans for kindergartens and school curricula.<br>▪ Resources and implementation:<br>– Continue to promote and implement health-promoting structures in schools.<br>▪ Knowledge and skills:<br>– Carry out health impact assessments, especially in the area of children and young people, before measures are implemented or rolled out.<br><br>**Sustainable food system**<br>▪ Policy and networking:<br>– Adapt the legal framework to avoid food waste (especially in retail and mass catering).<br>▪ Resources and implementation:<br>– Ensure healthy nutrition, especially for children and young people, including free, organic and balanced meals in schools and kindergartens.<br>▪ Knowledge and skills:<br>– Transparency and information on production chains, plant and animal nutrition and the effects on the climate and health (for example, food labelling).<br><br>**Resources for health promotion**<br>▪ Policy and networking:<br>– Develop a nationwide strategy for quality and funding guidelines in health promotion.<br>▪ Resources and implementation:

Country<br>Information Source	Implementation action(s), agency(ies) and key performance indicator(s)
	– Provide a budget for research in the field of health promotion.<br>▪ Knowledge and skills:<br>– Create a chair for health promotion and public health.<br><br>**Participation of population**<br>▪ Policy and networking:<br>– Promote political commitment to participation at federal and state level (establishment of appropriate structure).<br>▪ Resources and implementation:<br>– Set up a central overview of participation opportunities and experiences (online portals to map the needs and moods of the citizens).<br>▪ Knowledge and skills:<br>– Promote participatory research and raising awareness among researchers to new, needs-based issues (accompanying and methodological research, (pilot) projects, research funding).<br><br>**Health literacy and info**<br>▪ Policy and networking:<br>– Dissemination of health information in places where people are (such as the post office, bank and inn).<br>▪ Resources and implementation:<br>– Provide easily accessible information on counselling and placement services.<br>▪ Knowledge and skills:<br>▪ Promote media literacy and in dealing with social media, information abundance and fake news.
**Canada**<br>Creating a Healthier Canada: Making Prevention a Priority A Declaration on Prevention and Promotion from Canada's Ministers of Health and Health Promotion/Healthy Living (6)	None identified.
**England**<br>Public Health Outcomes Framework (PHOF) (7)	Two overarching indicators were identified:<br>▪ increased healthy life expectancy<br>▪ reduced differences in life expectancy and healthy life expectancy between communities.<br><br>4 further domains and associated indicators were outlined. 3 indicators for each domain are outlined below.

Country Information Source	Implementation action(s), agency(ies) and key performance indicator(s)
	**1. Improving the wider determinants of health**<br>Indicators: children in low income families, school readiness and pupil absence.<br>**2. Health Improvement**<br>Indicators: prescribing of long-acting reversible contraception, under 18 conceptions and maternity.<br>**3. Health Protection**<br>Indicators: fraction of mortality attributable to particulate air pollution, new sexually transmitted infection diagnoses and population vaccination coverage (children aged < 5 years old).<br>**4.Healthcare public health and preventing premature mortality**<br>Indicators: infant mortality, proportion of 5 year old children with dental decay and mortality rate from causes considered preventable.
**Finland**<br>Promoting well-being, health and safety in 2030: Decision in principle of the Government (8)	The Public Health Advisory Board monitors the implementation plan's measures and agrees on the monitoring indicators used to evaluate the achievement of the goals. The consultation committee annually summarises the progress of the measures and the implementation plan is updated based on this. In connection with the update, a hearing and a round of comments will be held, where proposals from stakeholders to promote implementation and possible new measures will be gathered.<br><br>1 sub-goal for each focal point, along with their associated strategic actions (A), timeline (T), coordinating body or lead (L) and cooperation partners (P) is outlined below.<br><br>**1. Opportunity for everyone to participate:**<br>People feel that they are equal and included when:<br>▪ they have the opportunity to make a sufficient living and they do not experience poverty:<br>- *A1: Reform social security*<br>T: 2020-2027<br>L: Social and Health Ministry (STM)<br>P: Ministry of Economic Affairs and Employment, Treasury (VM), Ministry of Education and Culture (OKM), Social Insurance Institution of Finland<br><br>**2. Good everyday environments:**<br>A good everyday environment for people is healthy, safe, barrier-free and aesthetically pleasing when it:<br>▪ encourages action to promote one's own well-being and health and supports the ability to study, work and function:<br>- *A43: municipalities encourage residents to be active for activities and hobbies through community and housing planning and experiencing a good and aesthetic environment*<br>T: 2020–no end point specified<br>L: Union of Municipalities<br>P: Municipalities, resident associations, VM, OKM, organisations, entrepreneurs and entrepreneur organisations, Art Promotion Center

Country<br>Information Source	Implementation action(s), agency(ies) and key performance indicator(s)
	**3. Activities and services that promote well-being and health:**<br>People need activities that promote well-being, health and safety, as well as high-quality and equal services. This means that:<br>▪ promoting well-being, health and safety is part of municipalities and regional management, operation and cooperation:<br>- *A96: form and describe structures supporting cooperation and coordination of the promotion of well-being, health and safety*<br>T: 2020-2022<br>L: STM<br>P: Municipalities, future welfare areas, regional state administrative agency, social competence centers, provinces<br><br>**4. Effectiveness with decision making:**<br>▪ decision makers promote well-being, health and safety and reduce inequality throughout the country, regionally and locally:<br>- *A130: strengthen the use of pre-assessment of the effects of decisions at the national, regional and local level by increasing awareness, understanding and know-how of the possibilities of using pre-assessment in the preparation of decision-making*<br>T: to be specified<br>L: Department of Health and Welfare<br>P: Municipalities, provinces, ministries, Confederation of Municipalities<br><br>**Key performance indicators**<br>Sotkanet.fi, are located within the Finnish institute for health and welfare and provide statistical information on welfare and health in Finland. They produce over 3,500 indicators on health, welfare and functioning of the service-system.(17)
**Northern Ireland**<br>Making life better: A whole system strategic framework for public health (9)	For each of the 6 themes long-term outcomes have been set with strategic supporting actions and commitments for the 2013 – 2015 period. 1 action along with its associated commitment is outlined below along with key indicators for each theme.<br><br>Key overarching indicators:<br>▪ Life expectancy: Differential between Northern Ireland (NI) average and most disadvantaged areas for men and women.<br>▪ Healthy life expectancy: Healthy Life Expectancy between NI average and most disadvantaged areas for men and women.<br>▪ Disability Free Life Expectancy: Disability Free Life Expectancy between NI average and most disadvantaged areas for men and women.<br><br>**Theme 1: Giving each child the best start**<br>Key indicators:<br>▪ Infant Mortality: Number of children dying before their first birthday per 1,000 live births<br>▪ Smoking During Pregnancy: Proportion of mothers smoking during pregnancy in NI and the most disadvantaged areas<br>▪ Breastfeeding: Proportion of mother’s breastfeeding on discharge and differential between NI average and most deprived.<br>▪ Educational Attainment – Key Stage 2: Proportion of primary pupils achieving at the expected levels in Key Stage 2 assessment in Communication and Using Mathematics<br>▪ Educational Attainment – General Certificate of Secondary Education (GCSE): Proportion of school leavers achieving at least 5 GCSEs at A*-C or equivalent, including GCSE English and Maths.

Country Information Source	Implementation action(s), agency(ies) and key performance indicator(s)
	**1. Good quality parenting and family support**<br>Actions and Commitments 2013 - 2015<br>*A) Promote and support positive parenting:*<br>▪ establishment of Family Support Hubs and systematic expansion of a range of initiatives and evidence based parenting support programmes.<br><br>**2. Healthy and confident children and young people**<br>Actions and Commitments 2013 - 2015<br>*A) Ensure high quality public health and social care services are provided for all children and young people, from ante natal care onwards to include:*<br>▪ the full range of health protection, health promotion, surveillance and screening and immunisation programmes.<br><br>**3. Children and young people skilled for life**<br>Actions and Commitments 2013 - 2015<br>*A)* Through implementation of "Every School a Good School" and the Literacy and Numeracy strategy:<br>▪ increase the proportion of primary pupils achieving at the expected level in Key Stage 2 in both Communication and Using Maths.<br><br>**Theme 2: Equipped throughout life**<br>Key indicators:<br>▪ Unemployment – Long Term: proportion of unemployed that have been unemployed for one year or longer<br>▪ Unemployment – Portion of 16 – 24 year olds who are not in education, employment, or training<br><br>**4. Ready for adult life**<br>Actions and Commitments 2013 - 2015<br>*A) Provide young people with access to:*<br>▪ careers information advice and guidance as required, to enable them to make effective career/learning choices.<br><br>**5. Employment, life-long learning and participation**<br>Actions and Commitments 2013 - 2015<br>*A)* Contribute to rising levels of employment by supporting the promotion of 25,000 jobs by 2015 as set out in the NI Economic Strategy.<br><br>**6. Healthy active ageing**<br>*A)* Improve job outcomes by providing temporary work for those aged 50+ who are unemployed and claiming benefit through the Steps to Work – Step Ahead 50+.

Country<br>Information Source	Implementation action(s), agency(ies) and key performance indicator(s)
	**Theme 3: Empowering health living**<br>Key indicators:<br>▪ Proportion of adults (aged 18 and over) who smoke and proportion in the most deprived areas<br>▪ Standardised rate for alcohol-related admissions in NI and the most disadvantaged areas<br>▪ Proportion of adults who drink above the sensible drinking guidelines suggested, and proportion in the most disadvantaged areas<br>▪ The teenage birth rate for mothers under the age of 17 – NI and most deprived areas<br>▪ Percentage of adults surveyed classified as obese, and proportion in the most disadvantaged areas<br>▪ Percentage of children surveyed classified as obese<br>▪ Mean Warwick Edinburgh Mental Wellbeing Scale by deprivation quintile<br>▪ Crude suicide Rate in NI and the most disadvantaged areas<br>▪ Number of patients with established hypertension and percentage of GP registered patients with established hypertension<br>▪ Number of people with one or more long term condition attending structured patient education/self-management programmes.<br><br>**7. Improved health and reduction in harm**<br>Actions and Commitments 2013 - 2015<br>*A) Develop and implement strategies, action plans and targeted programmes to:*<br>▪ reduce the number of people who:<br>– smoke<br>– are overweight or obese<br>– drink above the recommended alcohol limits<br>– misuse drugs.<br><br>**8. Improved mental health and wellbeing, and reduction in self-harm and suicide**<br>Actions and Commitments 2013 - 2015<br>*A)* Develop new policy to promote positive mental health, reduce self-harm and suicide.<br><br>**9. People are better informed about health matters**<br>Actions and Commitments 2013 - 2015<br>*A) Empower people to make healthier choices and informed decisions about their health by improving health literacy. This will include:*<br>▪ providing appropriate and accessible health information (making greater use of modern communication technology) and advice to all, which is evidence informed and tailored to meet specific needs, and which:<br>– encourages more people to present with early symptoms of health problems to HSC services<br>– promotes self-care, and sign-posts to appropriate support through, for example patient education/self-management programmes.<br><br>**10. Prevention embedded in services**

Country Information Source	Implementation action(s), agency(ies) and key performance indicator(s)
	A) Increase the emphasis on prevention and early intervention in the commissioning and delivery of Primary, Community, and Secondary Care services including:<br>▪ health professionals, particularly within primary care and Emergency departments, trained and encouraged to undertake substance misuse brief interventions and suicide prevention/mental health promotion intervention programmes across NI).<br><br>**Theme 4: Creating the Conditions**<br>Key indicators:<br>▪ Amount invested in public health<br>▪ Percentage of individuals in low-income groups before housing costs<br>▪ Percentage of children in low income groups before housing costs<br>▪ Proportion of the working-age population that is not in the labour force<br>▪ Proportion of social housing dwellings classified as non decent homes.<br>▪ Annual mean concentration level of Nitrogen Dioxide at urban background sites and urban roadside sites; Annual mean concentration level of particulate matter; Annual mean concentration level of Benzo(a) pyrene at monitored sites; Annual number of ozone breaches (days) at monitored sites<br>▪ Annual percentage compliance of Water Utility Sector Waste Water Treatment Works; Annual percentage mean zonal compliance of drinking water quality.<br><br>**11. A decent standards of living**<br><u>Actions and Commitments 2013 - 2015</u><br>*A)* Increase employment and prosperity for all by delivering the commitments set out in the NI Economic Strategy.<br><br>**12. Making the most of the physical environment**<br><u>Actions and Commitments 2013 - 2015</u><br>*A) Protect and promote good health and wellbeing through:*<br>▪ improving air quality to achieve objectives and targets established to protect health, and alerting those more likely to be affected when levels of air pollution are high.<br><br>**13. Safe and healthy homes**<br><u>Actions and Commitments 2013 - 2015</u><br>*A)* Deliver 8,000 social and affordable homes as set out in the PFG.<br><br>**Theme 5: Empowering Communities**<br>Key indicators:<br>▪ Social Capital: Proportion of respondents having volunteered in the past year<br>▪ Road Collisions: Number Killed or Seriously Injured casualty numbers per capita

Country<br>Information Source	Implementation action(s), agency(ies) and key performance indicator(s)
	**14. Thriving communities**<br>Actions and Commitments 2013 - 2015<br>*A) Strengthen and promote thriving communities which are welcoming, accessible and safe, and which support social inclusion through:*<br>▪ the Urban Regeneration and Community Development Policy framework which sets out clear priorities through policy objectives and supporting actions for operational programmes (includes targeted action for disadvantaged and areas at risk).<br><br>**15. Safe communities**<br>Actions and Commitments 2013 - 2015<br>*A)* PCSPs work collaboratively with the community and relevant agencies at local level and deliver Community Safety programmes so that people feel safer, have reduced fear of crime and increased confidence.<br><br>**16. Safe and healthy workplaces**<br>Actions and Commitments 2013 - 2015<br>A) Support more businesses to provide workplace health and wellbeing programmes to secure:<br>▪ improved physical and mental wellbeing.<br><br>**Theme 6: Developing Collaboration**<br>**17. A strategic approach to public health**<br>Actions and Commitments 2013 - 2015<br>*A)* Establish governance, implementation, engagement and monitoring arrangements at strategic, regional and local levels which interconnect to create a whole system approach.<br><br>**18.Strengthened collaboration for health and wellbeing**<br>Actions and Commitments 2013 - 2015<br>*A)* Maximise opportunities to strengthen local collaboration through the joint working arrangements between PHA and local government, and the outworking of local government reform and the new statutory duty of Community Planning process.
**Portugal**<br>National Health Plan (PNS) 2021-2030. Sustainable Health: from everyone to everyone (10)	**Intervention strategies for sustainable health (taking into account the main groups of health needs identified):**<br><br>**Investing: promoting and protecting health**<br>Reinforcing and/or implementing promotion strategies that health services evaluated as being more cost-effective. Establishing the analysis from systematic review of policies and intervention strategies of the different sectors, regarding the impact on health. Strategies and initiatives include: promotion of human rights; promotion of health literacy; boosting health-promoting environments and prevention and control of environmental risks.<br><br>**Include: Universal Health Coverage**

Country Information Source	Implementation action(s), agency(ies) and key performance indicator(s)
	It is necessary to continue to ensure the reinforcement and implementation of strategies that have been shown to be effective in reducing health needs. This includes needs which arise from high magnitude health problems, improving their efficiency, whether that is resulting from health problems of low magnitude and high risk potential, which to date are controlled in Portugal. Strategies and initiatives include: Universal health coverage (SNS and health for all, leaving no one behind); access to digital health: SNS24, telehealth and telemedicine; Integrated approach to patients' journeys, ensuring the transition of care modifiable risk factors (for example the treatment of hypertension and dyslipidemia, prescription of physical activity and healthy eating, brief interventions and Support Program Intensive).<br><br>**Innovate**<br>It is essential to promote knowledge of health determinants and on the effectiveness of intervention strategies through health research focused on sustainable health and health inequalities. The use of data enhances better quality information and a higher level of disaggregation. Incorporating scientific knowledge and good practice is encouraged in the strategy. The strategy aims to develop projects and a culture of digital transition, centred on citizens and including the Recovery and Resilience Plan. Actions include: establishment, maintenance or reinforcement of internal and external partnerships; promotion of knowledge and partnerships with academia and supporting a research agenda aligned with sustainable health.<br><br>**Prepare and anticipate the future**<br>Regarding the health needs arising from health problems currently of low or null magnitude but with an accelerated risk of emerging or re-emerging, it is important to clearly define and prioritise the strategies that ensure surveillance functions and emergency preparedness and response planning in public health. This should be integrated into a One Health approach. Actions include: continuous development / adjustment of plans, on a prospective basis, in order to quickly adapt intervention strategies to “new” health needs, but also to “instrumental areas” such as human resources, infrastructure and information and communication technologies; adaptation of health promotion and protection strategies according to the needs and expectations of the population and investment in patient safety (including the prevention of “new” resistance to antimicrobials, the avoidance of polypharmacy, especially in the elderly, and the excessive use of medication of the mental health forum, among others).<br><br>*Health objectives related to health needs arising from high magnitude problems set for 2030, in Portugal:*<br>1. Reduce the standardised premature mortality rate (less than 75 years old) from all causes of death in both sexes to 315 per 100,000 population.<br><br>*Health objectives related to health problems of low magnitude and high risk potential* set for 2030, in Portugal*<br>27. Ensure a maternal mortality rate equal to or less than 7.1 per 100,000 live births, in the three-year period 2028-2030 (average value of the three-year period with the best performance in Portugal in the last 6 years with available values).<br><br>**Further monitoring indicators include:**<br>▪ Perinatal mortality rate (per 1,000 live births and stillbirths over 28 weeks)<br>▪ Age-standardised premature preventable death rate (per 100,000 population)<br>▪ Age-standardised premature treatable mortality rate (per 100,000 population)<br>▪ 5-year survival of malignant tumour of the larynx, trachea, bronchi and lung (%)

Country<br>Information Source	Implementation action(s), agency(ies) and key performance indicator(s)
	▪ 5-year survival of malignant breast tumour (%).
**Scotland**<br>▪ National Performance Framework (NPF) (11)	The NPF tracks Scotland's progress in meeting its national outcomes. Each of the 11 National Outcomes has a set of indicators that underpin them. Performance is assessed as improving, maintaining or worsening based on the change between the last 2 data points of an indicator. The assessment of performance is made objectively and impartially by senior analysts in the Scottish Government. Decisions on performance are made independently of Scottish Government Ministers. 1 indicators for each outcome (along with their associated description is presented below).<br><br>**Outcome 1. Children and young people**<br>▪ Child social and physical development: measures the percentage of children with a concern at their 27-30 month review (as a % of children reviewed)<br>**Outcome 2. Communities**<br>▪ Perceptions of local area: percentage of adults who rate their neighbourhood as a very good place to live.<br>**Outcome 3. Culture**<br>▪ Attendance at cultural events or places of culture: percentage of adults who have attended or visited a cultural event or place in the last 12 months.<br>**Outcome 4. Economy**<br>▪ Productivity: Scotland's Rank for productivity against key trading partners in the Organisation for Economic Co-operation and Development.<br>**Outcome 5. Education**<br>▪ Work place learning: the percentage of employees who received on the job training in the last 3 months.<br>**Outcome 6. Environment**<br>▪ Visits to the outdoors: proportion of adults making one or more visits to the outdoors per week.<br>**Outcome 7. Fair work and business**<br>▪ The number of businesses: total number of private sector businesses (registered for Value Added Tax and or Pay As You Earn) in Scotland per 10,000 adults.<br>**Outcome 8. Health**<br>▪ Journeys by active travel: the proportion of short journeys less than 2 miles that are made by walking and the proportion of journeys<br>**Outcome 9. Human rights**<br>▪ Quality of public services: percentage of respondents who are fairly or very satisfied with the quality of local services (local health services, local schools and public transport).<br>**Outcome 10. International**<br>▪ A positive experience for people coming to live in Scotland: intended to measure one important dimension of migrants' experiences in Scotland – a strong sense of belonging.<br>**Outcome 11. Poverty**<br>▪ Relative Poverty after Housing Costs: proportion of individuals living in private households with an equivalised income of less than 60% of the UK median after housing cost.<br><br>A number of actions are outlined for each Priority along with their associated leads and or partners. 1 action for each Priority are outlined below.

Country<br>Information Source	Implementation action(s), agency(ies) and key performance indicator(s)
▪ Public Health Priorities for Scotland (12)	**Priority 1: A Scotland where we live in vibrant, healthy and safe places and communities:** partnership activity is underway through the Health and Justice Collaboration Improvement Board and specifically between, integration authorities and emergency services to better understand demand, to identify and support vulnerable people and to drive the prevention agenda. The Scottish Government and COSLA will seek to build further partnerships around data of this sort.<br>**Priority 2: A Scotland where we flourish in our early years:** public services are working with partners in the third and private sectors to focus on the early years and Getting It Right for Every Child continues to be the approach to improving outcomes and supporting the wellbeing of children and young people.<br>**Priority 3: A Scotland where we have good mental wellbeing:** implementation of the Scottish Government's 2017-27 Mental Health Strategy, which recognises the broad range of factors required to collectively improve wellbeing.<br>**Priority 4: A Scotland where we reduce the use of and harm from alcohol, tobacco and other drugs:** local Government, alcohol and drug partnerships, integration authorities, Police Scotland, the Scottish Prison Service and community planning partnerships are all developing locally tailored approaches to issues faced on the ground.<br>**Priority 5: A Scotland where we have a sustainable, inclusive economy with equality of outcomes for all:** Scotland's Economic Strategy places Inclusive Growth as a core priority.<br>**Priority 6: A Scotland where we eat well, have a healthy weight and are physically active:** the 2017-18 Programme for Government committed the Scottish Government to progress measures to limit the marketing of products high in fat, sugar and salt which disproportionately contribute to ill health and obesity and to deliver new services to support people with, or at risk of, type-2 diabetes, to lose weight. It set out the aspiration to increase physical activity levels and tackle diet and obesity in Scotland. It included commitments to boost investment in walking and cycling and put active travel at the heart of transport planning and to publishing a new Active Scotland Delivery Plan.
**Spain**<br>Public Health Strategy (ESP) 2022: Improving the Health and Well-Being of the Population (13)	Strategic line (SL), leads, actions (A) and associated indicators were outlined. One strategic line and action, and one indicator associated is provided below.<br><br>Strategic Line 1: Strengthening public health to improve the health of the population<br>**Lead:** the leadership of the Ministry of Health is essential to ensure coordination, cooperation and the establishment of alliances between the different Public Administrations with competencies in this area.<br><br>**SL1 – A1: Establish effective mechanisms for public health governance and cross-cutting health coordination in all policies.**<br>▪ SL1-A1.1: set up an inter-ministerial commission within the General State Administration to promote health in all policies, which may have working groups in specific areas.<br>Indicators associated with SL1 – A1:<br>▪ *Indicator (SL1 – A1- I1):* Inter-ministerial commission to promote health in all policies.<br><br>**SL1-A2: enhance the Spanish presence and participation in international decision-making forums related to public health and strengthen international collaboration with low- and middle-income countries**

Country Information Source	Implementation action(s), agency(ies) and key performance indicator(s)
	Indicator associated with SL1 – A2:<br>▪ *Indicator (SL1 – A2- I1):* Spanish participation in international public health-related institutions.<br><br>**SL1-A3: establish a State Centre for Public Health**<br>Indicators associated with SL1 – A3:<br>▪ *Indicator (SL1 – A3- I1):* State Centre for Public Health.<br><br>**SL1-A4: promote the evaluation of health impact of policies**<br>▪ SL1-A4.1: Develop the methodology for the evaluation of health impact of policies as reflected in Law 33/2011, 4 October, General Public Health, and promote an advisory network coordinated with the autonomous communities of Spain (CC.AA) to facilitate the evaluation of health impact of non-health interventions and the health approach in all policies.<br>Indicator associated with SL1 – A4:<br>▪ *Indicator (SL1 – A4- I1):* Methodology for the evaluation of health impact of policies.<br><br>**SL1-A5: strengthen public health services across Spain**<br>▪ SL1-A5.1: update the Portfolio of common public health services in Annex I of Royal Decree 1030/2006, 15 September, establishing the portfolio of common services of the National Health System and the procedure for its updating.<br>Indicators associated with SL1 – A5:<br>▪ *Indicator (SL1 – A5- I1):* Portfolio of common public health services.<br><br>**SL1-A6: standardise the choice of public health actions on a systematic basis based on the best scientific evidence, best practices and a portfolio of common public health services**<br>Indicators associated with SL1 – A6:<br>▪ *Indicator (SL1 – A6- I1):* Systematisation of choice of public health actions.<br><br>**SL1-A7: implement a human resources policy in public health that guarantees the generation and retention of talent, generational change, the appropriate size of human resources and the territorial deployment necessary to face present and future challenges with effectiveness and quality**<br>▪ SL1-A7.1: analyse the human resources needs in public health to guarantee the generation and retention of talent, generational replacement, and the appropriate size of the workforce for the functions performed.<br>Indicators associated with SL1 – A7:<br>▪ *Indicator (SL1 – A7- I1):* Working Group for Human Resources in Public Health Policy.<br><br>**SL1-A8: implement a public health training policy**<br>▪ SL1-A8.1: consensus on the core competencies that public health personnel need to master in order to respond to the performance of public health functions.

Country Information Source	Implementation action(s), agency(ies) and key performance indicator(s)
	Indicators associated with SL1 – A8:<br>▪ *Indicator (SL1 – A8- I1):* Core and optimal competencies for public health work.<br><br>**SL1-A9: strengthen research and innovation in public health.**<br>▪ SL1-A9.1: promote health research, with a public health and territorial cohesion perspective, within the framework of the Spanish Strategy for Science, Technology and Innovation 2021-2027, specifically the ISCIII's Strategic Action in Health, as well as the State's future scientific research strategies.<br>Indicators associated with SL1 – A9:<br>▪ *Indicator (SL1 – A9- I1):* National public health research map.<br><br>**SL1-A10: strengthen public health communication and advocacy**<br>▪ SL1-A10.1: develop, on a collaborative basis, a common public health communication strategy, including the design of a procedure for public dissemination of public health outcomes, the establishment of partnerships with formal (media) and informal stakeholders (such as social influencers and social media) and the availability of the necessary resources.<br>Indicators associated with SL1 – A10:<br>▪ *Indicator (SL1 – A10- I1):* Common public health communication strategy.<br><br>Strategic Line 2: Update public health surveillance and ensure response capacity to health risks and emergencies<br>**Lead:** not specified.<br><br>**SL2-A1: develop and implement the Public Health Surveillance Strategy**<br>▪ SL2-A1.1: public health surveillance is a system that timely integrates information from all sources and structures necessary to respond to public health information needs.<br>Indicator associated with SL2 – A1:<br>▪ *Indicator (SL2 – A1- I1):* Compliance with the Public Health Surveillance Strategy.<br><br>**SL2-A2: improve monitoring and management of vaccination programmes.**<br>▪ SL2-A2.1: develop an integrated national vaccine register system interoperable at European level, in collaboration with the CC.AA.<br>Indicators associated with SL2 – A2:<br>▪ *Indicator (SL2 – A2- I1):* Vaccination and Immunisation Information System.<br><br>**SL2-A3: improve the response to public health threats at local, regional, national and international levels.**<br>▪ SL2-A3.1: develop the Early Warning and Rapid Response System of the State Public Health Surveillance Network, integrated into the Public Health Surveillance System, ensuring coordination between all agencies and stakeholders to ensure adequate early detection and rapid response to public health alerts.<br>Indicators associated with SL2 – A3:

Country<br>Information Source	Implementation action(s), agency(ies) and key performance indicator(s)
	▪ *Indicator (SL2 – A3- I1):* Development of the Early Warning and Rapid Response System of the State Public Health Surveillance Network.<br><br>Strategic Line 3: Improve the health and well-being of the population through disease prevention and the promotion of healthy lifestyles and healthy, safe and sustainable environments<br>**Lead:** not specified.<br><br>**SL3-A1: promote and encourage healthy and sustainable food.**<br>▪ SL3-A1.1: collaborate intersectorally and at all levels of Public Administration to promote lifelong healthy eating in all settings (with special emphasis on education, health and work) and to encourage people to make healthy and sustainable food choices.<br>Indicators associated with SL3 – A1:<br>▪ *Indicator (SL3 – A1- I1):* Breastfeeding.<br><br>**SL3-A2: encourage and promote physical activity and reduce sedentarism.**<br>▪ SL3-A2.1: collaborate in an intersectoral, interdisciplinary way and at all levels of Public Administration to inform and raise awareness among the population about health-enhancing physical activity.<br>Indicators associated with SL3 – A2:<br>▪ *Indicator (SL3 – A2- I1):* Information and awareness-raising on health enhancing physical activity.<br><br>**SL3-A3: promote policies/initiatives aimed at reducing the use of tobacco, alcohol and other substance and non-substance related addictions.**<br>▪ SL3-A3.1: collaborate with the Government Delegation for the National Drugs Plan in the implementation of the National Strategy on Addictions 2017-2024 and its Action Plans, with regard to tobacco, alcohol and the use of, and addiction to, other psychoactive substances, as well as the addictive potential of other behaviours (such as gambling and screen time).<br>Indicators associated with SL3 – A3:<br>▪ *Indicator (SL3 – A3- I1):* Action Plan on Addictions 2021-2024.<br><br>**SL3-A4: promote sexual health from a positive, comprehensive and inclusive approach.**<br>▪ SL3-A4.1: carry out training, education and promotion actions on comprehensive sexual health aimed at the population (with special emphasis on the adolescent and youth population and those in situation of vulnerability).<br>Indicators associated with SL3 – A4:<br>▪ *Indicator (SL3 – A4- I1):* Comprehensive sexual health training, education and promotion.<br><br>**SL3-A5: promote disease prevention.**<br>▪ SL3-A5.1: develop a national vaccination strategy and improve vaccination coverage for immunopreventable diseases.<br>Indicators associated with SL3 – A5:<br>▪ *Indicator (SL3 – A5- I1):* National Vaccination Plan.

Country Information Source	Implementation action(s), agency(ies) and key performance indicator(s)
	**SL3-A6: promote safe environments for all.**<br>▪ SL3-A6.1: to inform and raise awareness among citizens, professionals and decision-makers about prevention of unintentional injuries and violence.<br>Indicators associated with SL3 – A6:<br>▪ *Indicator (SL3 – A6- I1):* Information and awareness-raising on prevention of unintentional injuries and violence.<br><br>**SL3-A7: promote a healthier environment.**<br>▪ SL3-A7.1: implement the Strategic Plan for Health and Environment (PESMA) and the action programmes arising there from, with a health in all policies and One Health approach.<br>Indicators associated with SL3 – A7:<br>▪ *Indicator (SL3 – A7- I1):* PESMA.<br><br>**SL3-A8: promote food security interventions and programmes.**<br>▪ SL3-A8.1: collaborate intersectorally and at all levels of the Public Administration in the monitoring of the National Plan for the Official Control of the Food Chain 2021-2025 with the Spanish Agency for Food Safety and Nutrition, and provide the necessary information to evaluate results.<br>Indicators associated with SL3 – A8:<br>▪ *Indicator (SL3 – A8- I1):* National Plan for the Official Control of the Food Chain 2021-2025.<br><br>**SL3-A9: promote healthy, safe and sustainable educational environments.**<br>▪ SL3-A9.1: promote in the educational environment (infant, primary and secondary) specific health promotion itineraries that incorporate basic and advanced knowledge on healthy, safe and sustainable lifestyles or behaviours.<br>Indicators associated with SL3 – A9:<br>▪ *Indicator (SL3 – A9- I1):* Network of Health Promoting Schools in Spain.<br><br>**SL3-A10: encourage the local environment to promote health and well-being.**<br>▪ SL3-A10.1: promote health, health equity, community participation and assets for health through coordination between primary care, public health, municipalities and other supra-municipal local bodies, neighbourhoods and citizens.<br>Indicators associated with SL3 – A10:<br>▪ *Indicator (SL3 – A10- I1):* Health and community participation.<br><br>**SL3-A11: to provide a working environment that ensures the safety and protection of people's health and well-being.**<br>▪ SL3-A11.1: implement the Spanish Strategy for Health and Safety at Work 2022- 2027 and as many actions that could be established within the framework of the National Commission for Health and Safety at Work; as well as the regional strategies in this area.<br>Indicators associated with SL3 – A11:

Country<br>Information Source	Implementation action(s), agency(ies) and key performance indicator(s)
	▪ *Indicator (SL3 – A11- I1):* Spanish Strategy for Health and Safety at Work 2022-2027.<br><br>**SL3-A12: promote public health actions in the healthcare environment and social services.**<br>▪ SL3-A12.1: develop community care policies and programmes for health promotion, prevention and early detection of diseases and health problems from primary care in collaboration with public health.<br>Indicators associated with SL3 – A12:<br>▪ *Indicator (SL3 – A12- I1):* Comprehensive Lifestyle Counselling Programmes.<br><br>**SL3-A13: controlling public health at borders.**<br>▪ SL3-A13.1: modernise foreign health by drawing up a Strategic Plan for foreign health that includes digitalisation and improvements in the quality of its services, among others, as well as its regulatory framework, all with the collaboration and agreement of the Ministry of Territorial Policy.<br>Indicators associated with SL3 – A13:<br>▪ *Indicator (SL3 – A13- I1):* Strategic Plan for the modernisation/improvement of foreign health.<br><br><u>Strategic Line 4: Promoting population health and health equity throughout the life course</u><br>**Lead:** not specified.<br><br>**SL4-A1: encourage the protection and promotion of an active and healthy childhood and adolescence.**<br>▪ SL4-A1.1: collaborate intersectorally and at all levels of Public Administrations to tackle the childhood obesity pandemic.<br>Indicators associated with SL4 – A1:<br>▪ *Indicator (SL4 – A1- I1):* Protection from violence in the child and adolescent population.<br><br>**SL4-A2. Encourage the protection and promotion of active and healthy ageing.**<br>▪ SL4-A2.1: collaborate intersectorally and from all levels of Public Administrations to promote active and healthy ageing, and good treatment (WHO Decade of Healthy Ageing 2020-2030, among others).<br>Indicators associated with SL4 – A2:<br>▪ *Indicator (SL4 – A2- I1):* Protection from violence in the elderly.<br><br>**SL4-A3. Ensure that health policies promote equitable addressing of the needs of all.**<br>▪ SL4-A3.1: collaborate intersectorally and at all levels of Public Administrations to promote equity in the health and well-being of the population (National Health Equity Strategy, among others).<br>Indicators associated with SL4 – A3:<br>▪ *Indicator (SL4 – A3- I1):* National Health Equity Strategy.<br><br>A number of **Main Indicators** were also outlined:

Country<br>Information Source	Implementation action(s), agency(ies) and key performance indicator(s)
	▪ Life expectancy at birth<br>▪ Healthy life years<br>▪ Health status of the population **(**Percentage of people aged 15 and over describing their health status as good or very good)<br>▪ Deaths by cause of death<br>▪ Mortality attributed to cardiovascular disease, cancer, diabetes or chronic respiratory diseases<br>▪ Suicide mortality rate<br>▪ Limitations on activities of daily living (Percentage of persons indicating limitations on activities of daily living in the last 6 months according to sex and age group. Population aged 15 and over)<br>▪ Limited mobility (Percentage of people indicating difficulty in mobility according to sex and age group. Population aged 15 and over)<br>▪ Emotional well-being (Percentage of people indicating moderate or severe depressive symptomatology according to sex and age group. Population aged 15 and over).
**Sweden**<br>Towards good and equal health: A framework for implementing and monitoring the national Public Health policy (15)	No implementation actions were identified.<br>An original set of indicators was proposed to monitor health inequalities, social determinants of health, and the health status of the population using a life-course perspective.(18) This included 130 target area indicators and 50 health indicators.(19) This was then narrowed to a set of core indicators for monitoring target areas. These were either performance indicators (PI) or structure indicators (SI), and were to indicate if health differed between different groups in the population and if differences increased or decreased overtime. The indicators are according to a number of reporting groups such as gender, age, level of education, income and country of birth, to follow whether inequality is increasing or decreasing. Authorities responsible for issues within each target area were also outlined. 1 focus area and indicator has been outlined below for each target area.<br><br>**Target Area 1. Conditions in early life:**<br>▪ equal maternal and child health care: *PI: risky use of alcohol when enrolling in maternal healthcare*<br>**Example of authorities responsible for important issues in this target area:** the Swedish Social Insurance Agency, the Swedish Agency for Family Law and Parental Support and the Sami School Board.<br><br>**Target Area 2. Knowledge, skills and education/training:**<br>▪ a good learning environment at school: *SI: teachers with a teaching university degree in elementary school*<br>**Example of authorities responsible for important issues in this target area:** the Swedish Board of Student Finance, the Swedish Agency for Higher Vocational Education and the National Board of Health and Welfare.<br><br>**Target Area 3. Work, working conditions and work environment:**<br>▪ to have a job: *PI: unemployment; PI: young people who neither work nor study*<br>**Example of authorities responsible for important issues in this target area:** the Swedish Public Employment Service, the Swedish Work Environment Authority and the Swedish Board of Student Finance.<br><br>**Target Area 4. Income and economic resources:**

Country<br>Information Source	Implementation action(s), agency(ies) and key performance indicator(s)
	▪ distribution of income in the population: *SI/PI: economic standard, percentiles, median*<br>**Example of authorities responsible for important issues in this target area:** the Employment Service, the Social Insurance Agency and the Authority for Family Law and Parental Support.<br><br>**Target Area 5. Housing and neighbourhood conditions:**<br>▪ access to a fully functional and affordable home: *SI: municipalities deficit on housing for certain groups in vulnerable situations; PI: overcrowding*<br>**Example of authorities responsible for important issues in this target area:** the Swedish Agency for Marine and Water Management, the Swedish Prison and Probation Service and the Swedish Environmental Protection Agency.<br><br>**Target Area 6. Health behaviours:**<br>▪ Limit the availability of products harmful to health: *PI: daily tobacco smoking, 16-84 years; PI: risk consumption of alcohol, 16-84 years*<br>**Example of authorities responsible for important issues in this target area:** the Swedish National Council for Crime Prevention, the Public Health Agency of Sweden, the Swedish Consumer Agency.<br><br>**Target Area 7. Control, influence and participation:**<br>▪ equal participation in democracy: *SI: voting in general elections; PI: trust in society's institutions*<br>**Example of authorities responsible for important issues in this target area:** the Swedish Crime Victim Compensation Agency, the Discrimination Man and the Swedish National Council for Popular Education.<br><br>**Target Area 8. Equitable and health-promoting health and medical services:**<br>▪ accessibility to meet different needs: *PI: refusal of medical care despite perceived need*<br>**Example of authorities responsible for important issues in this target area:** the Public Health Agency of Sweden**,** the National Agency for Education and the National Board of Health and Welfare.<br><br>**Cross-sectoral authorities relevant to all target areas:** the Ombudsman for Children; the Non-Discrimination Ombudsman; the Swedish Gender Equality Agency; the Agency for Participation and the Swedish Agency for Youth and Civil Society.<br><br>The 21 county administrative boards are state authorities that must work to ensure that national goals have an impact in the county, taking regional conditions and conditions into account. They do not have substantive responsibility like the authorities above, but must work across sectors and coordinate various societal interests based on a state-wide perspective and the efforts of state authorities within their area of responsibility. The county boards are thus a link between the municipal and the state level.<br><br>**A number of health outcomes/indicators were also specified:**<br>▪ remaining average life expectancy<br>▪ premature mortality

Country<br>Information Source	Implementation action(s), agency(ies) and key performance indicator(s)
	▪ self-assessed general state of health<br>▪ overall measure of mental health<br>▪ overall measure of morbidity or good health.<br><br>A more detailed assessment of a number of conditions was also proposed such as mental health, diseases of the circulatory system and cancer.

Table 10. Stakeholder engagement methods used in the development of national public health strategies of selected countries.

**Country**<br>**Information Source**	**Stakeholder engagement**
**Australia**<br>National Preventive Health Strategy: Valuing health before illness: Living well for longer (3)	**Public consultation methods**<br>▪ online public survey<br>▪ online public consultations on the consultation paper and the draft strategy<br>▪ findings of other relevant health consultations conducted by the Australian Government in recent years.<br>**Targeted consultation methods**<br>▪ Expert Steering Committee guided strategy development. Committee was composed of experts in public health, research, health promotion, medical, allied health, nursing and consumer advocacy fields.<br>▪ Targeted consultations carried out to hear the views of experts in different fields of prevention, community representatives, consumer groups, advocacy organisations, and the public about what is important to keep themselves, their families and their communities healthy.
**Austria**<br>▪ Austrian Health Targets – long healthy life years for all (4)<br><br>▪ ROADMAP "Future Health Promotion": 10 packages of measures for a health-promoting future in Austria (5)	**Public consultation methods**<br>▪ Online public consultations conducted in May to August 2011 and April 2012 to provide the public with an opportunity to contribute ideas on the topic of "health maintenance”.<br>**Targeted consultation methods**<br>▪ Group involving 40 representatives from various political and social areas established from October 2011 to March 2012. The group developed a proposal for health goals for the Federal Health Commission in 5 workshops. Group representatives also fed back results to their organisations in order to ensure broad acceptance of the goals among these organisations.<br>**Other engagement methods**<br>▪ Electronic newsletter circulated to interested parties from May to August 2011 to inform them of developments during the process<br>▪ Health goals presented at Federal Health Conference in May 2012.<br><br>**Public consultation methods**<br>▪ Public survey and supplemental focus groups conducted in July 2021 to coincide with 10 years of health goals in Austria. Topics addressed included everyday health, the COVID-19 pandemic and personal health resources<br>▪ Online citizen dialogue carried out from July to September 2022 to enable interested citizens to contribute their experiences of needs so that health promotion measures could be targeted to where they are needed most<br>▪ Three citizen councils took place between September and October 2022, in which randomly selected citizens from all over Austria attended a 1.5-day event to intensively discuss the future of health promotion and to develop 5 core messages with concrete proposals for measures.<br>**Targeted consultation methods**

Country<br>Information Source	Stakeholder engagement
	▪ 'Foresight process' consisting of 3 events (launch, scenario and vision forum) took place in 2021. Involved 150 experts and stakeholders working together to develop visionary goals for the further development and sustainable anchoring of health promotion by 2050 in different areas of society.<br>▪ Online consultation with experts carried out from May to June 2022 to enable experts to recommend starting points and concrete measures for various areas of society, building on previous processes and existing strategies.<br>▪ Four focus groups and one discussion round conducted between August and September 2022 to enable active participation in consultation by groups that were less able to participate via the other methods, such as people affected by poverty, young people, people with a migratory background, senior citizens, and people with health restrictions and their family carers.<br>**Other engagement methods**<br>▪ Future Health Promotion Forum took place in October 2022, attended by 153 experts and other stakeholders. The results of all groups were presented and discussed in line with existing national and international strategies in order to develop needs-based and effective bundles of measures.
**Canada**<br>Creating a Healthier Canada: Making Prevention a Priority A Declaration on Prevention and Promotion from Canada's Ministers of Health and Health Promotion/Healthy Living (6)	None identified.
**England**<br>Public Health Outcomes Framework (PHOF) (7)	None identified.
**Finland**<br>Promoting well-being, health and safety in 2030: Decision in principle of the Government (8)	**Targeted consultation methods**<br>▪ After the end of the Health 2015 programme, statements were requested from government ministries to enable them to contribute to and propose changes to the decision in principle.<br>▪ Five stakeholder workshops took place in autumn 2020, in which stakeholders contributed to the implementation plan. This included specifying measures, identifying partners for various measures, and expressing views on how to utilise the plan. Attendees included representatives of universities, universities of applied sciences and other research and development organisations, representatives of organisations, representatives of municipalities and municipal associations, representatives of trade unions and institutions under ministries.<br>▪ In total, more than 200 experts participated in the development of the decision in principle and implementation plan.
**Northern Ireland**<br>Making life better: A whole system strategic framework for public health (9)	**Public consultation methods**<br>▪ A proposed new 10-year public health framework, 'Fit and Well – Changing Lives 2012-2023', was published for consultation from mid-July 2012 to mid-November 2012.<br>**Targeted consultation methods**

Country<br>Information Source	Stakeholder engagement
	▪ In 2012, the Department engaged with a number of network organisations and partnerships to seek the views of key stakeholder sectors and population groups. Including reports by the network organisations, a total of 141 responses were received.<br>▪ Following the consultation process, two cross-sectoral workshops were held in 2013 to consider the feedback received on 'Fit and Well – Changing Lives' and to explore how this should influence the final framework.<br>**Other engagement methods**<br>▪ At the same time as the consultation, the NI Assembly Health Committee conducted an inquiry into health inequalities which took evidence from a range of expert witnesses nationally and internationally. Findings from the report of this inquiry were factored into framework development.
**Portugal**<br>National Health Plan (PNS) 2021-2030. Sustainable Health: from everyone to everyone (PNS 2021-2030) (10)	A co-creation process was adopted as the overarching approach to strategy development.<br><br>**Public consultation methods**<br>▪ Public consultation conducted from April 2022 to May 2022, with 114 entities and citizens from different sectors of society participating.<br>**Targeted consultation methods**<br>▪ The co-creation process involved over 100 stakeholders from inside and outside the health sector during the development of the strategy. Two groups were established and engaged with throughout the strategy development process:<br>o Monitoring Commission, which included representatives from relevant sectors<br>o Advisory Board, comprising of approximately 40 individuals with recognised relevant technical and or scientific knowledge and present or past experience.<br>▪ Distinct engagement methods used as part of the co-creation process included:<br>o Multisectoral seminar in October 2019 to begin the development of the National Health Plan.<br>o Online survey of Monitoring Commission representatives to identify and prioritise perceived health needs and to elicit opinions on the extent to which health needs were being or could be affected by the COVID-19 pandemic.<br>o Mixed-methods health goals setting exercise undertaken with experts from the National Health Programs to set goals based on the results of the online survey.<br>o The selection of intervention strategies was carried out based on contributions from the Monitoring Commission. An instrument for collecting their contributions was constructed from a matrix of all intervention strategies under implementation or planned in National Health Programs.
**Scotland**<br>▪ National Performance Framework (NPF) (11)<br><br>▪ Public Health Priorities for Scotland (12)	▪ None identified.<br><br>**Public consultation methods**<br>▪ Regional engagement events which involved several hundred people from across the public and third sectors.<br><br>**Targeted consultation methods**

**Country**<br>**Information Source**	**Stakeholder engagement**
	▪ Collaborative work with public health and other experts to develop criteria, and to assess and weigh the evidence.<br>▪ Testing of emerging conclusions with experts and other stakeholders.
**Spain**<br>Public Health Strategy (ESP) 2022: Improving the Health and Well-Being of the Population (13)	**Targeted consultation methods**<br>▪ Contributions sought from CC.AA through the Public Health Commission and through discussion of the draft in the Public Health Commission and in CISNS<br>▪ The Spanish Society for Public Health and Health Administration produced the Conceptual and Methodological Support Report for the Public Health Strategy, with information within this strategy used in ESP 2022<br>▪ Consultation with Units of the Ministry of Health and other departments<br>▪ Scientific societies, professionals and experts were involved in reviewing drafts and providing multiple contributions.
**Sweden**<br>Towards good and equal health: A framework for implementing and monitoring the national Public Health policy (15)	**Targeted consultation methods**<br>▪ Strategic dialogues led by the Director General of the Public Health Agency. Dialogues took place with authorities with substantive responsibility for areas related to the policy targets, authorities with relevant cross-sector responsibilities, and local and regional governments. The purposes of the dialogues were to discuss a possible support structure for public health work and to ensure the quality of the work.<br>▪ An external expert group contributed to the work. This group consisted of academics and experts in public health and strategy with scientific and methodological knowledge, knowledge about equal health and existing indicators to monitor public health, and knowledge of the situation from local, regional and national perspectives.

Bibliography

1. World Health Organisation (WHO). 21st Century Health Challenges: Can the Essential Public Health Functions Make a Difference? Discussion Paper [Internet]. Geneva: World Health Organization (2021). Available from: https://apps.who.int/iris/bitstream/handle/10665/351510/9789240038929-eng.pdf?sequence=1&isAllowed=y.
2. Sian Griffiths. Public Health Functions. In: Detels R, Gulliford M, Karim QA, Tan CC, editors. *Oxford Textbook of Global Public Health*. 6th ed. Oxford: Oxford University Press (2015). p. 1544.
3. Australian Government Department of Health and Aged Care. National Preventative Health Strategy: Valuing Health before Illness: Living Well for Longer [Internet]. Canberra: Australian Government (2021) [cited 2023 May 31]. Available from: https://www.health.gov.au/sites/default/files/documents/2021/12/national-preventive-health-strategy-2021-2030_1.pdf.
4. Federal Ministry of Health and Women’s Affairs Austria. Health Targets Austria - Relevance – Options – Contexts: Abbreviated Version [Internet]. Vienna: Federal Ministry of Health and Women’s Affairs (2017). Available from: https://gesundheitsziele-oesterreich.at/website2017/wp-content/uploads/2017/05/health-targets-austria.pdf.
5. Agenda for Health Promotion Austria. Path to a Better Quality of Life: Roadmap "Future Health Promotion" [Internet]. Vienna: Health Austria (2023) [2023]. Available from: https://agenda-gesundheitsfoerderung.at/kozug/roadmap.
6. Ministers of Health and Health Promotion/Healthy Living Canada. Creating a Healthier Canada: Making Prevention a Priority: A Declaration on Prevention and Promotion from Canada’s Ministers of Health and Health Promotion/Healthy Living [Internet]. Ottawa: Public Health Agency of Canada (2010). Available from: https://www.canada.ca/content/dam/phac-aspc/migration/phac-aspc/hp-ps/hl-mvs/declaration/pdf/dpp-eng.pdf.
7. Public Health England. Public Health Outcomes Frameworks 2019-2022: At a Glance [Internet]. London: Public Health England (2019). Available from: https://assets.publishing.service.gov.uk/government/uploads/system/uploads/attachment_data/file/862264/At_a_glance_document2.pdf.
8. Ministry of Social Affairs and Health F. Promoting Well-Being, Health and Safety in 2030: Decision in Principle of the Government [Internet]. Helsinki: Ministry of Social Affairs and Health (2021). Available from: https://stm.fi/documents/1271139/20825107/Hyvinvoinnin+terveyden+ja+turvallisuuden+edist%C3%A4minen+2030+-+valtioneuvoston+periaatep%C3%A4%C3%A4t%C3%B6s_F2020.pdf/66e90eeb-138e-b6b3-9b68-83232c174240/Hyvinvoinnin+terveyden+ja+turvallisuuden+edist%C3%A4mine?t=1592395038000.
9. Department of Health Northern Ireland. Making Life Better: A Whole System Strategic Framework for Public Health 2013 -2023 [Internet]. Northern Ireland: Department of Health (2014). Available from: https://www.health-ni.gov.uk/sites/default/files/publications/dhssps/making-life-better-strategic-framework-2013-2023_0.pdf.
10. Director General of Health Portugal. National Health Plan 2021-2030. Sustainable Health: From Everyone to Everyone (Pns 2021-2030) [Internet]. Lisbon: Ministry of Health (2023). Available from: https://pns.dgs.pt/files/2022/12/PNS2021-2030_FINAL-para-Edicao.pdf.

11. Scottish Government. National Performance Framework [Internet]. Scotland: Scottish Government (2023). Available from: https://nationalperformance.gov.scot/.
12. Scottish Government. Scotland's Public Health Priorities [Internet]. Scotland: Scottish Government (2018) [updated 2018 June 14]. Available from: https://www.gov.scot/publications/scotlands-public-health-priorities/pages/1/.
13. Ministry of Health Spain. Public Health Strategy 2022: Improving the Health and Well-Being of the Population Madrid: Ministry of Health (2022). Available from: https://www.sanidad.gob.es/ciudadanos/pdf/Public_Health_Strategy_2022_Pending_NIPO.pdf.
14. Ministry of Health and Social Affairs Sweden. Good and Equal Health – a Developed Public Health Policy Prop. 2017/18:249 [Internet]. Sweden: Government of Sweden (2018) [updated 2018 April 20]. Available from: https://www.regeringen.se/rattsliga-dokument/proposition/2018/04/prop.-201718249.
15. Public Health Agency of Sweden. Towards a Good and Equitable Health: A Framework for Implementing and Monitoring the National Public Health Policy [Internet]. Sweden: Public Health Agency of Sweden (2021). Available from: https://www.folkhalsomyndigheten.se/contentassets/bb50d995b033431f9574d61992280e61/towards-good-equitable-health.pdf.
16. Federal Ministry of Health and Women's Affairs Austria. Health Promotion Strategy within the Framework of the Federal Target Management Agreement [Internet]. Vienna: Federal Ministry of Health and Women's Affairs (2021). Available from: https://www.sozialministerium.at/Themen/Gesundheit/Gesundheitssystem/Gesundheitsreform-(Zielsteuerung-Gesundheit)/Gesundheitsf%C3%B6rderungsstrategie-im-Rahmen-des-Bundes-Zielsteuerungsvertrags.html.
17. Finnish Institute for Health and Welfare. Data Content [Internet]. Helsinki: Finnish Institute for health and welfare (2023). Available from: https://sotkanet.fi/sotkanet/en/data/indicators.
18. Public Health Agency of Sweden. Core Indicators for Follow-up of the Public Health Policy Objective (2022) [13 June 2023]. Available from: https://www.folkhalsomyndigheten.se/publikationer-och-material/publikationsarkiv/k/karnindikatorer-for-uppfoljning-av-det-folkhalsopolitiska-malet/.
19. Public Health Agency of Sweden. Towards a Good and Equitable Health – a Framework for Implementing and Monitoring the National Public Health Policy: Attachment 1: List of Indicators for Monitoring Health Status and Social Determinants of Health [Internet]. Sweden: Public Health Agency of Sweden (2021). Available from: https://www.folkhalsomyndigheten.se/globalassets/publicerat-material/publikationer/artikelnr-21273/attachment-1-indicators-used-monitoring-health-status-social-determinants-health-21273-1.pdf.
